# Model-free extraction of spin label position distributions from pseudocontact shift data†
†Electronic supplementary information (ESI) available. See DOI: 10.1039/c6sc03736d



**DOI:** 10.1039/c6sc03736d

**Published:** 2017-01-20

**Authors:** Elizaveta A. Suturina, Daniel Häussinger, Kaspar Zimmermann, Luca Garbuio, Maxim Yulikov, Gunnar Jeschke, Ilya Kuprov

**Affiliations:** a School of Chemistry , University of Southampton , Highfield Campus , Southampton , SO17 1BJ , UK . Email: i.kuprov@soton.ac.uk; b Department of Chemistry , University of Basel , St. Johanns Ring 19 , CH-4056 Basel , Switzerland; c Department of Chemistry and Applied Biosciences , Swiss Federal Institute of Technology in Zurich , Vladimir Prelog Weg 1-5/10 , CH-8093 Zürich , Switzerland

## Abstract

A significant problem with paramagnetic tags attached to proteins and nucleic acids is their conformational mobility. Each tag is statistically distributed within a volume between 5 and 10 Angstroms across; structural biology conclusions from NMR and EPR work are necessarily diluted by this uncertainty. The problem is solved in electron spin resonance, but remains open in the other major branch of paramagnetic resonance – pseudocontact shift (PCS) NMR spectroscopy, where structural biologists have so far been reluctantly using the point paramagnetic centre approximation. Here we describe a new method for extracting probability densities of lanthanide tags from PCS data. The method relies on Tikhonov-regularised 3D reconstruction and opens a new window into biomolecular structure and dynamics because it explores a very different range of conditions from those accessible to double electron resonance work on paramagnetic tags: a room-temperature solution rather than a glass at cryogenic temperatures. The method is illustrated using four different Tm^3+^ DOTA-M8 tagged mutants of human carbonic anhydrase II; the results are in good agreement with rotamer library and DEER data. The wealth of high-quality pseudocontact shift data accumulated by the biological magnetic resonance community over the last 30 years, and so far only processed using point models, could now become a major source of useful information on conformational distributions of paramagnetic tags in biomolecules.

## Abstract 2

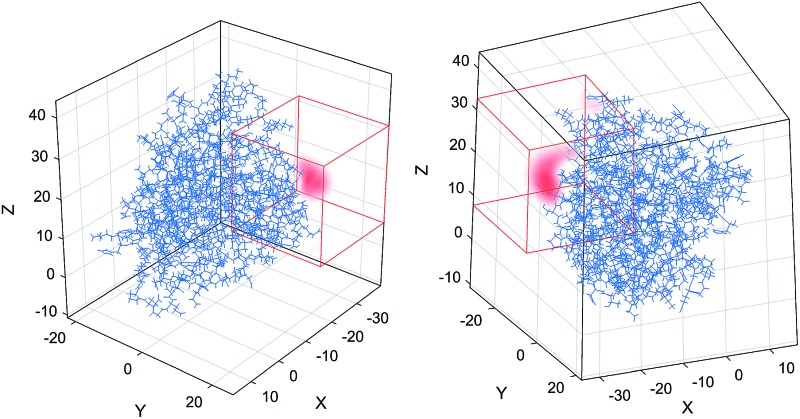
Not a point, but a cloud: advanced PCS data analysis using 3D probability density reconstruction provides more information.

## 1. Introduction

Pseudocontact shift (PCS) is an additional chemical shift caused by the presence of a rapidly relaxing paramagnetic centre near the nucleus.^1,2^ PCS is well understood theoretically^3–7^ and is widely employed as a source of structural restraints in metalloproteins,^8–10^ where commonly occurring Ca^2+^, Mg^2+^, Mn^2+^ and Zn^2+^ binding sites can often coordinate a lanthanide ion instead.^11^ A paramagnetic centre may also be introduced artificially by attaching a lanthanide ligand tag to the protein surface.^12,13^

The subject has a long-standing problem – lanthanide-containing protein tags have significant conformational mobility.^14^ Even DOTA-M8,^15^ which uses a sterically overcrowded – and therefore rigid – metal cage,^16^ still has a flexible linker. The conformational mobility of lanthanide tags is visible in the distance distributions measured by double electron resonance,^17^ and in molecular dynamics simulations.^18^ In this situation the commonly used point paramagnetic centre approximation^3,19^ for PCS is not expected to be valid,^14,20,21^ but quantum chemical calculations^6,7^ are prohibitively expensive.

The problem is solved by the recently discovered partial differential equation that treats the probability density of the metal ion and the resulting pseudocontact shift as scalar fields in three dimensions.^20^ We demonstrate here that it may be used to recover the spin label position distribution from the experimental PCS data. This creates a new window into protein structure and dynamics.

## 2. Extracting spin label distributions from PCS data

The point paramagnetic centre approximation for the pseudocontact shift^3,19^ *σ*(**r**) experienced by a nucleus at the position **r** relative to the metal has the following form:1
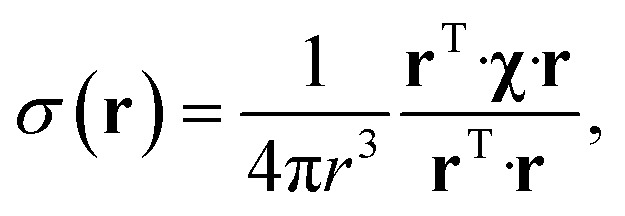
where **χ** is the traceless part of the magnetic susceptibility tensor. If the paramagnetic centre is distributed with some probability density *ρ*(**r**), the convolution of this density with eqn (1) obeys^21^ the partial differential equation that we have recently derived:^20^
2
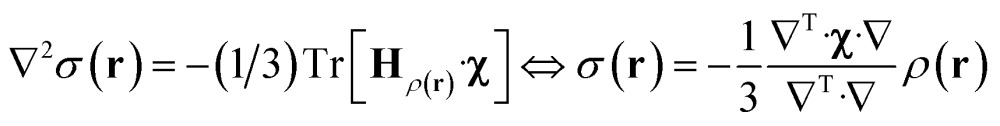
where ∇ is the gradient operator, **H**_*ρ*(**r**)_ is the Hessian of *ρ*(**r**) and the denominator is to be understood as the inverse Laplacian. The best way to solve this equation is to use fast Fourier transforms:^21^
3
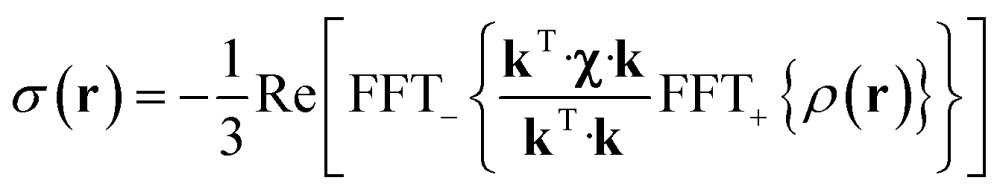



The task of recovering *ρ*(**r**) from point measurements of *σ*(**r**) at the nuclei may be formulated as finding the paramagnetic centre probability density that minimises the following functional:4
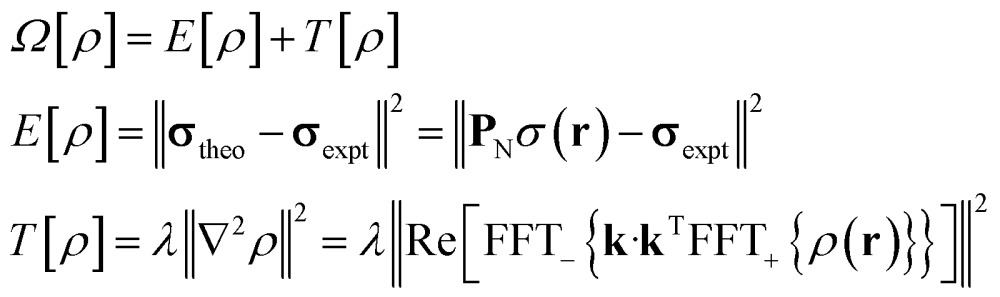
where *E*[*ρ*] is the least squares error relative to the experimentally measured pseudocontact shifts at the nuclear locations sampled by the operator **P**_N_, and *T*[*ρ*] is a Tikhonov regularisation term emphasizing smooth solutions with *λ* being the regularisation parameter selected using the L-curve method.^22^

State-of-the art numerical optimisation algorithms^23^ require first and second variations of the error functional with respect to the probability density. The first variations are:5
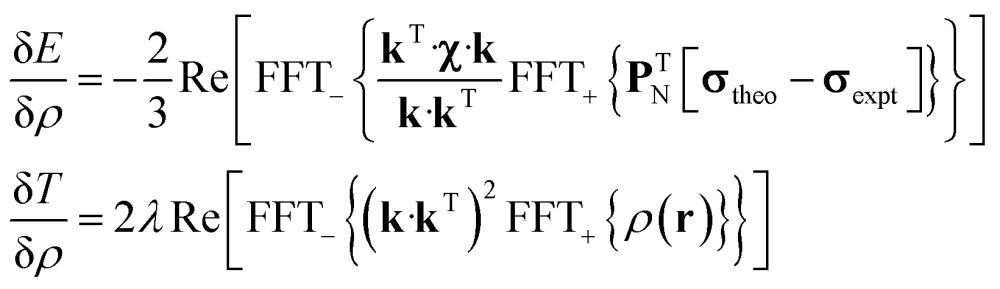
and the actions by the second variations on a probe function *η*(**r**) are:6
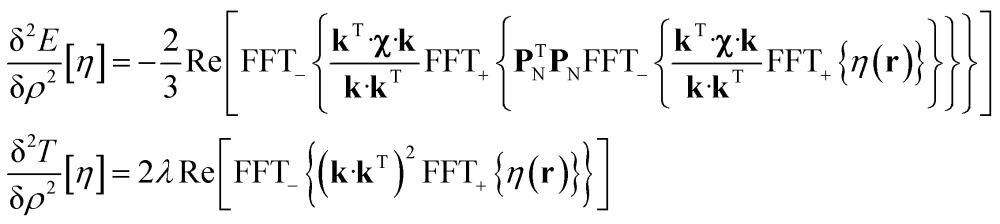



The trust region reflective Newton–Raphson minimiser, as implemented in the Optimisation Toolbox^24^ supplied with *Matlab*, was used to obtain the optimum paramagnetic centre probability density on a finite grid, subject to the non-negativity constraint. Numerical implementation details are discussed in our recent paper^21^ and the associated *Matlab* source code is available in the paramagnetic NMR module supplied with versions 1.8 and later of the *Spinach* library.^25^

## 3. Materials and methods

### 3.1 Protein preparation

The pACA plasmid used for the production of human carbonic anhydrase II (hCA-II) mutants was a generous gift from Carol A. Fierke (University of Michigan).^26^ Double and triple mutants were prepared by sequential site-directed mutagenesis (performed as recommended by Zheng *et al.* ^27^). All constructs were expressed in uniformly ^15^N labelled form and in selectively ^15^N-Leu labelled form; all expressions were carried out in BL21(DE3)pLysS competent cells using standard methods. Conjugation with Lu^3+^, Tm^3+^ or Gd^3+^ containing DOTA-M8 tags was performed as previously described.^15^

### 3.2 Nuclear magnetic resonance

Backbone assignment was performed using standard TROSY-enhanced triple-resonance experiments: HNCO, HNCA, HN(CO)CA, HN(CA)CO, and HNCACB. Residues 2–20, H64, I91, P155, G156, T199, T200 and P201 could not be assigned unambiguously.

Pseudocontact shifts were obtained by comparing ^1^H–^15^N HSQC spectra of diamagnetic Lu^3+^ and paramagnetic Tm^3+^ DOTA-M8 tagged mutants. PCS assignment was performed in two stages. At the first stage, at least eight of the 26 leucine peaks in the selectively ^15^N-Leu labelled mutants were identified manually and supplied to NUMBAT,^28^ along with ^15^N positions from the X-ray structure (PDB:; 3KS3) of hCA-II.^29^ This enabled the identification of all leucine ^15^N atoms and the extraction of the approximate point model parameters (metal position and the anisotropic part of the magnetic susceptibility tensor). At the second stage, these parameters were used in another round of NUMBAT calculations on the uniformly ^15^N labelled mutants to assist in the identification of the rest of the shifted signals, yielding a total of 364 (S217C), 366 (S50C, S220C) and 397 (S166C) unambiguous ^1^H and ^15^N PCS assignments. The relevant data is included into the ESI.†

### 3.3 Electron paramagnetic resonance

Pulsed EPR measurements were performed using a home-built Q-band EPR spectrometer^30^ equipped with a rectangular broad-band resonator that can accommodate oversized samples.^31,32^ EPR samples of the spin-labelled hCA-II were prepared as a mixture of equal volumes of glycerol and the protein solution in an aqueous phosphate buffer at pH 6.8; the resulting protein concentration was approximately 100 μM. 50 μL of this mixture was transferred into a thin-walled quartz tube with 2.9 mm outer diameter, flash-frozen by immersion into liquid nitrogen and stored at –80 °C.

Spin label distance distributions were measured at 10 K using the four-pulse DEER sequence.^33^ All pulses were 12 ns long; the frequency offset between the pump pulse and the detection pulse was 300 MHz. The duration of all DEER traces was at least 3.0 μs – long enough to sample and subtract the intermolecular background. Distance distributions were extracted using the *DeerAnalysis* package.^34^ The optimal values of the Tikhonov regularization parameter were found using the L-curve method.^22^

### 3.4 Rotamer library details

Position distributions for the lanthanide ion enclosed in the DOTA-M8 chelating tag^15^ were also estimated by constructing, for each of the four tagging sites (S50C, S166C, S217C, S220C), a complete set of distinct energetically accessible rotamers. The set was built by Monte-Carlo sampling of the dihedral angles present in the tag, followed by agglomerative clustering using the following distance metric:7
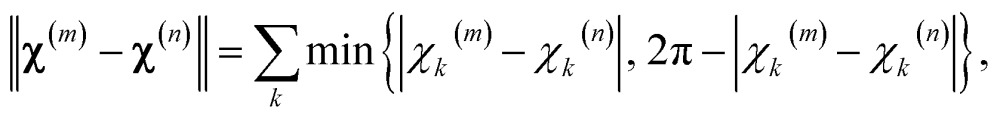
where **χ** denotes a set of dihedral angles, *m* and *n* indices enumerate Monte-Carlo ensemble members, and *k* runs over the elements of **χ**. An UPGMA agglomerative hierarchical clustering tree^35^ was built with this distance metric using *Matlab* Statistics and Machine Learning Toolbox.^36^ The initial structure for the DOTA-M8 tag attached to a cysteine side chain by a disulphide bond was generated using ZORA DFT B3LYP/SVP energy minimisation in *ORCA*.^37^ The bond lengths were then fixed and only the dihedral angles were varied, with the energies computed using the UFF model^38^ and populations estimated using the Boltzmann distribution.^39^ We followed the same approach as Joseph *et al.*,^40^ with softened Lennard-Jones potentials that account for the librational motion in the protein environment^41^ – the equilibrium inter-atomic distances were scaled down until convergence was achieved in the distance distributions. Direct inspection of the energy profiles along each of the dihedral angles in the DOTA-M8 linker identifies 2 × 2 × 2 × 3 × 3 × 3 × 3 × 3 = 1944 distinct energy minima; this was supplied as the target number of clusters to the agglomerative hierarchical clustering algorithm. Each of the resulting clusters was viewed as defining a distinct rotamer with dihedral angles defined as the Boltzmann average over the cluster. The population of each rotamer was defined as the sum of the Boltzmann weights of all cluster members. The resulting library was integrated into the *MMM* package.^41^ Rotamer populations for the DOTA-M8 tag bound to the protein were then computed as described by Polyhach *et al.* ^41^

## 4. Results and discussion

**Fig. 1:**
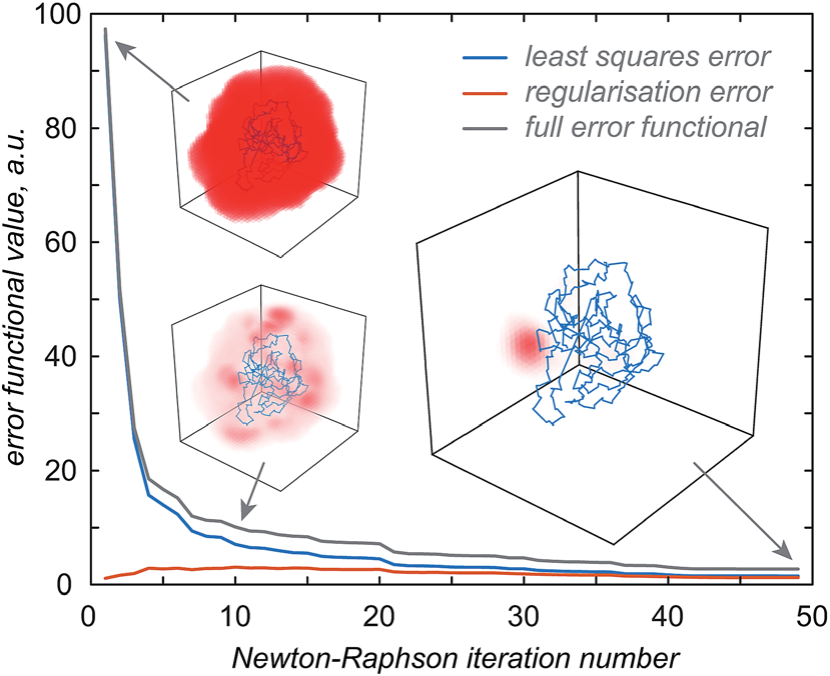
Evolution of the probability density of the Tm^3+^ ion attached to C220 of the S220C mutant of human carbonic anhydrase II with a DOTA-M8 tag during the error functional optimisation process. The initial guess is a uniform distribution within the volume that is at least 2.0 Å from all atoms of the protein itself and at most 12 Å from any of its atoms, corresponding to the region of the space realistically accessible by the Tm^3+^ ion in a tag attached anywhere on the protein surface. As the optimisation proceeds, the probability density gradually becomes zero in the locations that are not consistent with the experimental PCS data. At the end of the optimisation, the probability density is localised, subject to the standard accuracy conditions associated with Tikhonov regularisation,^22^ in the region of space actually accessible to the Tm^3+^ ion.

The first step in the paramagnetic centre probability density reconstruction is to find its approximate location. The process is illustrated graphically in Fig. 1. The initial guess for the probability density is a uniform distribution over all points in space that are realistically accessible to the tag – outside the van der Waals radius of the protein and no further than 12 Å from its surface. The initial guess for the magnetic susceptibility tensor is obtained from the point model fit. The initial localisation of the paramagnetic centre distribution is typically achieved in 50 Newton–Raphson iterations or fewer – about a minute on the wall clock when a *Tesla K40* coprocessor card is used to run the FFTs on a 128 × 128 × 128 point grid.

**Fig. 2:**
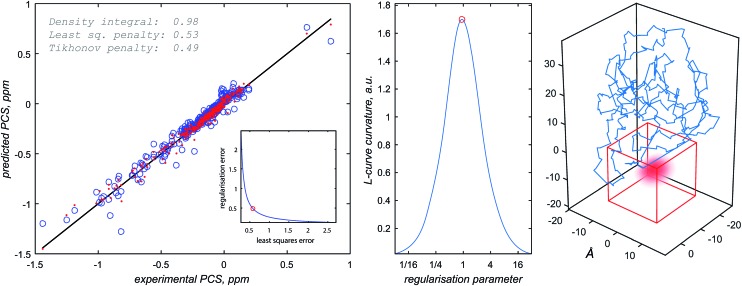
Diagnostic information and the outcome of a typical paramagnetic centre probability density reconstruction run. After the initial localisation stage (Fig. 1), the region of space in which the probability density is allowed to vary is chosen (right panel, red cube). Multiple reconstruction runs with different values of the regularisation parameter are performed to obtain the L-curve (left panel, cut-in). The optimum regularisation parameter is extracted as the maximum curvature point on the L-curve (middle panel). The final reconstruction is performed to obtain the probability density (right panel, red cloud) and the fitting plot (left panel). Blue circles in the left panel correspond to the point model fit and the red dots to the probability density fit.

Once the approximate location of the paramagnetic centre becomes clear, the refinement of its distribution on a finer grid can proceed with a much reduced variational volume that only involves the region of space immediately adjacent to the approximate location of the tag. An example of such a volume is given in Fig. 2 (red cube in the right panel); a 20 × 20 × 20 Å cube is in practice sufficient.

The optimisation is then performed repeatedly for different values of the regularisation parameter *λ* in eqn (4). The resulting L-curve is shown as an inset in the left panel of Fig. 2; the corresponding curvature plot is in the middle panel. The optimum value of the regularisation parameter (indicated with a red circle) is calculated and the optimisation is performed again with that value. This yields the paramagnetic centre probability density (red cloud in the right panel) on a fine grid, as well as the plot of the back-calculated pseudocontact shifts against the experimental ones (Fig. 2, left panel). A 256 × 256 × 256 point grid is in practice sufficient; the calculation takes a few hours on a *Tesla K40* card.

Once the draft probability density is obtained, a different least squares optimisation is run, this time with respect to the five independent elements of the effective magnetic susceptibility tensor. The tensor is updated and the probability density reconstruction procedure described above is repeated. The whole procedure is performed multiple times until self-consistency is achieved between **χ** and *ρ*(**r**).

The procedure described above relies on two significant assumptions. Firstly, the protein structure is treated as rigid and only the paramagnetic centre is assumed to be delocalised. This is an approximation – in a real protein structure the pseudocontact shifts are also averaged over the distributions in the nuclear positions. From NMR data, in well-defined structures these have position distributions within about 0.4 Å for backbone atoms and 1.0 Å for all heavy atoms.^42^ It is therefore to be expected that the paramagnetic centre distribution obtained from the PCS data would be broader than the real one by approximately that amount. Secondly, eqn (2)–(6) rely on the magnetic susceptibility tensor being the same at each point in the tag distribution. This is not necessarily true because the orientation of the tag can vary. This matter has recently been studied in detail by Shishmarev and Otting;^14^ their conclusion was that a single effective **χ** tensor can describe the PCS field reasonably well, even in the presence of significant tag mobility. A recent experimental study by Abdelkader *et al.* has also concluded that using an effective magnetic susceptibility tensor to mask its orientational distribution is a good approximation.^43^ The algebraic structure of eqn (2) suggests that local variations in **χ** can be compensated by local variations in the probability density – the practical consequences of the constant effective magnetic susceptibility tensor assumption are therefore minor ripples in the probability density. A technical analysis of the accuracy of this approximation is given in the ESI;† the conclusion is that the resulting uncertainty is multiplicative – it would never generate probability density where there was none; it can only scale the true density by a factor related to the norms of the susceptibility tensors involved.

**Fig. 3:**
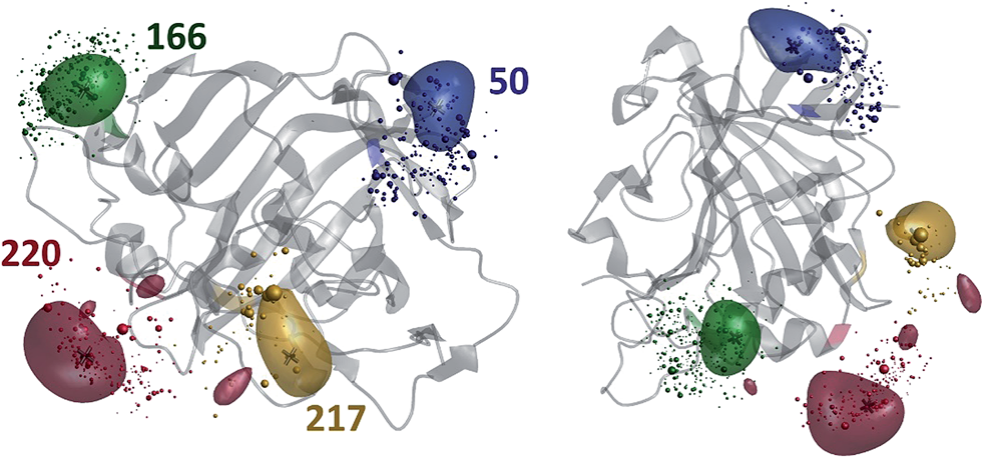
Tm^3+^ ion position distributions in DOTA-M8 tagged human carbonic anhydrase II, extracted from PCS data (the translucent coloured bubbles enclose 50% of the total probability) and overlaid with rotamer library predictions (swarms of coloured spheres with volumes proportional to the Boltzmann populations of the corresponding rotamers). The locations of Tm^3+^ ions predicted by the point model fits are indicated with dark grey three-dimensional crosses. The protein is visualized as a translucent grey ribbon model with the labelled positions (SER to CYS mutation followed by DOTA-M8 tagging) coloured.

**Fig. 4:**
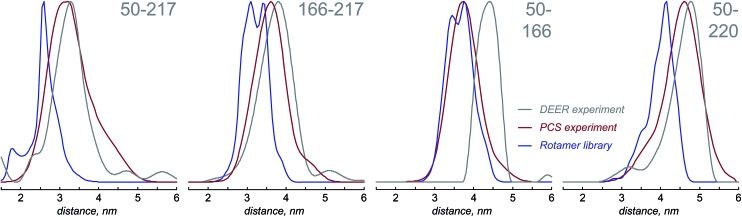
Distance distributions extracted from the PCS-based probability densities obtained in this work (red lines), compared with DEER-based distance distributions measured in the structurally identical Gd^3+^ tagged proteins (grey lines), compared with the same distributions extracted from the rotamer library results (blue lines).

The results of the paramagnetic centre probability density reconstructions for S50C, S166C, S217C and S220C mutants of hCA-II with a Tm^3+^ containing DOTA-M8 tag attached to the corresponding cysteines are presented in Fig. 3 and 4. As could be expected, the paramagnetic centre locations predicted by the point model fits (dark grey three-dimensional crosses in Fig. 3) are located close to the centroids of the probability density distributions computed by regularization (coloured translucent bubbles). The distributions also overlap significantly with the Tm^3+^ ion positions predicted by the rotamer library (swarms of coloured spheres), providing an independent experimental confirmation of the validity of the rotamer library approach.^40,41^

An important secondary contribution to the chemical shift in paramagnetic systems arises from the residual anisotropic chemical shifts (RACS) that are caused by the weak alignment of the magnetic susceptibility tensor by the applied magnetic field. This effect was recently studied in detail by Otting *et al.*, who estimated the RACS correction magnitude for backbone ^15^N nuclei to be about 0.1 ppm for a dysprosium ion rigidly coordinated inside a protein structure.^44^ In the context of this work, the RACS correction was assumed to be negligible – a thulium ion (weaker PCS than dysprosium) at the end of a flexible linker would generate a much smaller chemical shift correction than 0.1 ppm, which is itself smaller than the scatter observed in Fig. 2. In situations when RACS are suspected to be significant, we recommend running the reconstruction using only proton PCS data because the effect is negligible for protons.

Fig. 4 presents the comparison between the distance distributions between lanthanide ions in tags at different sites obtained using three physically different methods: DEER,^34^ PCS (this work) and rotamer libraries.^40,41^ For three out of four tagging site pairs, the agreement of the PCS data with the DEER data is very good and significantly better than the agreement of the rotamer library prediction with the DEER data. In the remaining case, both the rotamer library and the PCS prediction deviate from the DEER data by the same amount. This indicates that PCS-based reconstruction of the spatial distribution of the paramagnetic centre performs better than rotamer libraries, although testing on a broader range of proteins would be necessary to make that conclusion in a definitive way. One of the possible explanations for the difference between PCS and DEER reconstructions for the 50–166 dataset is the presence of structural changes caused by the double mutation – individual S50C and S166C mutations (used for PCS) might not have influenced the overall protein geometry in a detectable way, but in the double mutant (used for DEER) the changes could be significant. This conjecture is supported by the fact that a related 166–220 double mutant is completely unstable and could not be expressed in a non-degraded form. The observed difference should not therefore be held against either method; it yields a useful structural insight.

The question of tag probability density reconstruction is particularly pertinent to the many ongoing efforts to characterise domain mobility in proteins.^45–48^ Pseudocontact shift is a convenient parameter for those studies because the timescale of its emergence (*i.e.* the unpaired electron magnetisation equilibration time in a lanthanide) is in the picoseconds, and the time scale of its observation (*i.e.* the reciprocal frequency difference between the signals in the paramagnetic NMR spectrum) is in the milliseconds. The former is much faster than protein domain mobility, and the latter is much slower, meaning that the probability density is well defined and simply reflects structural heterogeneity – the corresponding theory is not troubled by the local dynamics effects that make nuclear spin relaxation theory so complicated.

The following would be a reasonable usage scenario for the method described above. In a multi-domain protein or nucleic acid, one of the domains should be tagged with a lanthanide. Pseudocontact shifts measured *in the same domain* should be used to run a probability density reconstruction. The resulting cloud would be a measure of how rigidly the tag is immobilised relative to its home domain. At the second stage, pseudocontact shifts measured *in the other domains* should be used to reconstruct the volume that is available to the tag; that volume is an indication of the volume explored by its home domain relative to other domains. The width of the tag distribution in its home domain would then be a measure of the uncertainty in the resulting conformational mobility conclusions. Rigidly immobilised and highly predictable tags^49^ are therefore likely to be beneficial.

On the detailed map of protein mobility analysis methods recently published by Ravera *et al.*,^50^ the PCS technique described in this paper belongs to the L-curve class, with a significant difference that the penalty functional is not molecular energy (of which there is no notion in the probability density formalism), but the more traditional Laplacian norm.^22^ There exists a possibility of introducing a contrast functional similar to the maximum entropy one,^20^ but we would not recommend using it because it is hard to justify on physical grounds, and also because the distance distribution widths are already in good agreement with other methods (Fig. 4).

## 5. Conclusions and future work

A probability density of the paramagnetic centre may be extracted from PCS data using the recently discovered partial differential equation for PCS^20,21^ and the Tikhonov regularisation method. The resulting technique is experimental in the same sense as DEER^33^ – it requires regularisation at the data processing stage – but it explores a very different range of conditions: a room-temperature solution rather than a glass at cryogenic temperatures. This makes it highly complementary to DEER because the difference between static structural heterogeneity at cryogenic temperatures and the dynamic structural ensemble at ambient conditions becomes easy to observe. The PCS method also has the advantage of only requiring NMR equipment, which is easier to come by than cryogenic pulsed EPR gear. In many cases it would not even need a chemically attached tag, since about 30% of all proteins coordinate metal ions naturally and can usually accommodate a variety of PCS-friendly ions.^8,51,52^ Some mobility is suspected to exist at those coordination sites;^21^ it would be an interesting target for further exploration using 3D reconstructions.

Because the extracted distributions have the physical meaning of probability densities, multiple independent datasets (for example, from different metals or different structures in a bundle) may be combined by multiplication. We did not explore this matter further, but it bears notice that the possibility exists.

The probability density reconstruction technique described above is also important because it provides an independent experimental validation for the DEER method – so far, the distributions of the tag at each labelling site could only be modelled, and no experimental technique was available to check the results, except for DEER itself. The good agreement on both the centres and the widths of the distance distributions shown in Fig. 4 is a strong endorsement of the two-electron dipolar spectroscopy results.
