# Supplementary material for: Model-free extraction of spin label position distributions from pseudocontact shift data
Source: Chem Sci. 2017 Jan 20;8(4):2751–7. doi: 10.1039/c6sc03736d (PMC5426344; doi:10.1039/c6sc03736d)
Supplement: Supplementary file 1 [file SC-008-C6SC03736D-s001.pdf]

Electronic Supplementary Material (ESI) for Chemical Science.
This journal is © The Royal Society of Chemistry 2017

Supporting Information for

"Model-free extraction of spin label position distributions from pseudocontact shift data"

Elizaveta A. Suturina[1], Daniel Häussinger[2], Kaspar Zimmermann[2], Luca Garbuio[3], Maxim Yulikov[3], Gunnar Jeschke[3], Ilya Kuprov[1,*]

[1]*School of Chemistry, University of Southampton, Highfield Campus, Southampton, SO17 1BJ, UK*

[2]*Department of Chemistry, University of Basel, St. Johanns Ring 19, CH-4056 Basel, Switzerland*

[3]*Department of Chemistry and Applied Biosciences, Swiss Federal Institute of Technology in Zurich, Vladimir Prelog Weg 1-5/10, CH-8093 Zürich, Switzerland*

[*]Corresponding author: i.kuprov@soton.ac.uk

1. Fitting PCS data using the point and the delocalised models

The outputs (theory *vs.* experiment) of the fitting using the delocalised model described in the main text is shown in Figure S1 below. The span of the PCS values differs for different mutants: the largest absolute PCS of 11 ppm is detected for S166C; in the case of S220C largest absolute PCS does not exceed 1.5 ppm. This is due to the different orientation of the susceptibility tensors in the two mutants, as shown in Table S1. The deviation from experiment visually appears to be larger for S220C, but that is a visual scaling effect – in fact the largest standard deviation for the delocalised model is found for S50C (Table S1).

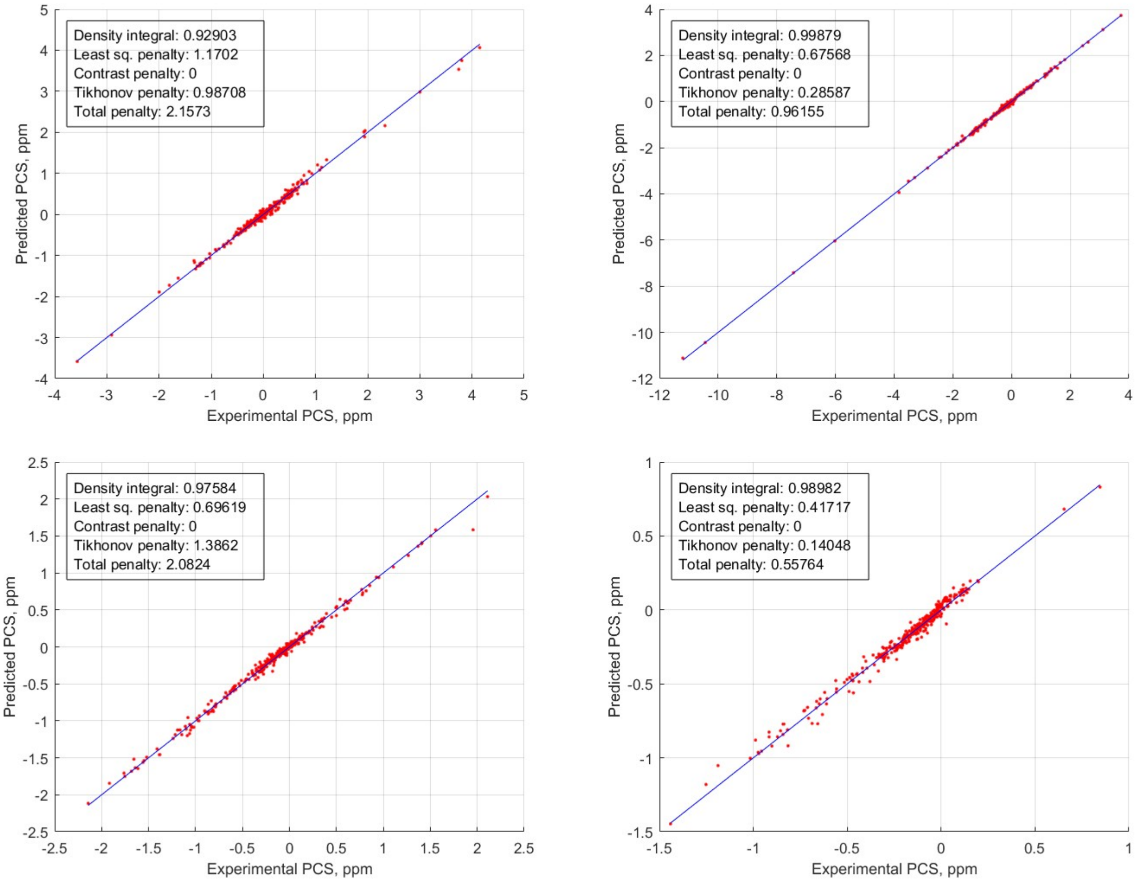

Figure S1. *Plots of theoretical vs. experimental values of the pseudocontact shift (in ppm) obtained by fitting using the delocalised model (see the main text for details) for human carbonic anhydrase II with a Tm(III) DOTA-M8 tag attached to the cysteine residue of S50C mutant (top left), S166C mutant (top right), S217C mutant (bottom left), and S220C mutant (bottom right).*

All assigned PCS values have been used for the fitting of susceptibility tensor. In Table S1 below, the eigenvalues of the traceless part of the susceptibility tensor are labelled such that $|\chi_z| > |\chi_y| > |\chi_x|$. In that notation, the axiality is $\chi_{ax} = 3/2\chi_z$, and the rhombicity is $\chi_{rh} = (\chi_x - \chi_y)/2$. The orientation is specified using Euler angles in the ZYZ convention. The standard deviation of the fitted values of the susceptibility tensor axiality and rhombicity do not exceed 5% of the corresponding value. In all cases the use of the delocalised model results in a reduction in the RMSD of the fit. The point model is unable to reproduce pseudocontact shifts on the nuclei positioned closer than about 15 Angstrom to the tag, and therefore produces larger RMSD values and significantly different susceptibility tensor parameters. The agreement of the point model with the distributed model improves when (as per the usual practice in the literature) the proximate nuclei are artificially excluded from the fit.

Table S1. Magnetic susceptibility tensor parameters (cubic Angstroms in SI units) extracted from PCS data fits using the point model, the point model with proximate nuclei excluded, and the distributed model (see the main text for details).

Mutant	Model	χ_{ax} (Å³)	χ_{rh} (Å³)	α (°)	β (°)	γ (°)	RMSD of the PCS fit (ppm)	Improvement in the RMSD
S50C	point	0.19	0.037	108	141	28	0.061	11%
	point (>15 Å³)	0.21	0.043	104	142	116		
	delocalised	0.25	0.056	108	143	214	0.054	
S166C	point	0.37	0.036	232	57	307	0.047	15%
	point (>15 Å³)	0.37	0.039	53	123	141		
	delocalised	0.36	0.039	232	58	304	0.040	
S217C	point	0.19	0.056	329	115	323	0.135	69%
	point (>15 Å³)	0.26	0.066	143	71	125		
	delocalised	0.27	0.071	323	108	145	0.042	
S220C	point	0.24	0.013	183	29	104	0.057	42%
	point (>15 Å³)	0.23	0.022	16	154	4		
	delocalised	0.30	0.018	183	31	129	0.033	

Table S2. Paramagnetic centre position (in Å) obtained from the point model fit of the PCS data. The standard deviations are below 0.25 Å in all cases.

Mutant	Model	x (Å³)	y (Å³)	z (Å³)
S50C	point	-27.2	13.3	18.8
	point (>15 Å³)	-27.5	13.6	18.2
S166C	point	-16.0	-3.6	-11.0
	point (>15 Å³)	-16.2	-3.6	-11.0
S217C	point	-23.8	-16.4	20.2
	point (>15 Å³)	-24.8	-17.5	19.6
S220C	point	-14.7	-26.3	4.1
	point (>15 Å³)	-13.0	-26.4	3.2

The following are the sources of deviations between the point model and the experimental PCS data:

1) The difference between the assumed protein structure and the real structural ensemble in solution.

2) Residual anisotropic chemical shifts (RACS) on the nitrogen nuclei (see **Table S3**).

3) The mobility of the paramagnetic tag.

The delocalised PCS model accounts for the dominant source of deviations (tag mobility), but does not address the other two sources. The RACS problem is easily solved by only fitting proton PCS data. Nothing can be done about the difference between the assumed and the "real" protein structure, but the same is true for any other method anywhere in structural biology.

The improvement in the PCS fitting at short distances achieved by the use of the delocalised model is illustrated in Figure S2. The key advantage of the delocalised model is that the data points that were previously discarded as a nuisance now yield useful information about the probability density of the paramagnetic tag.

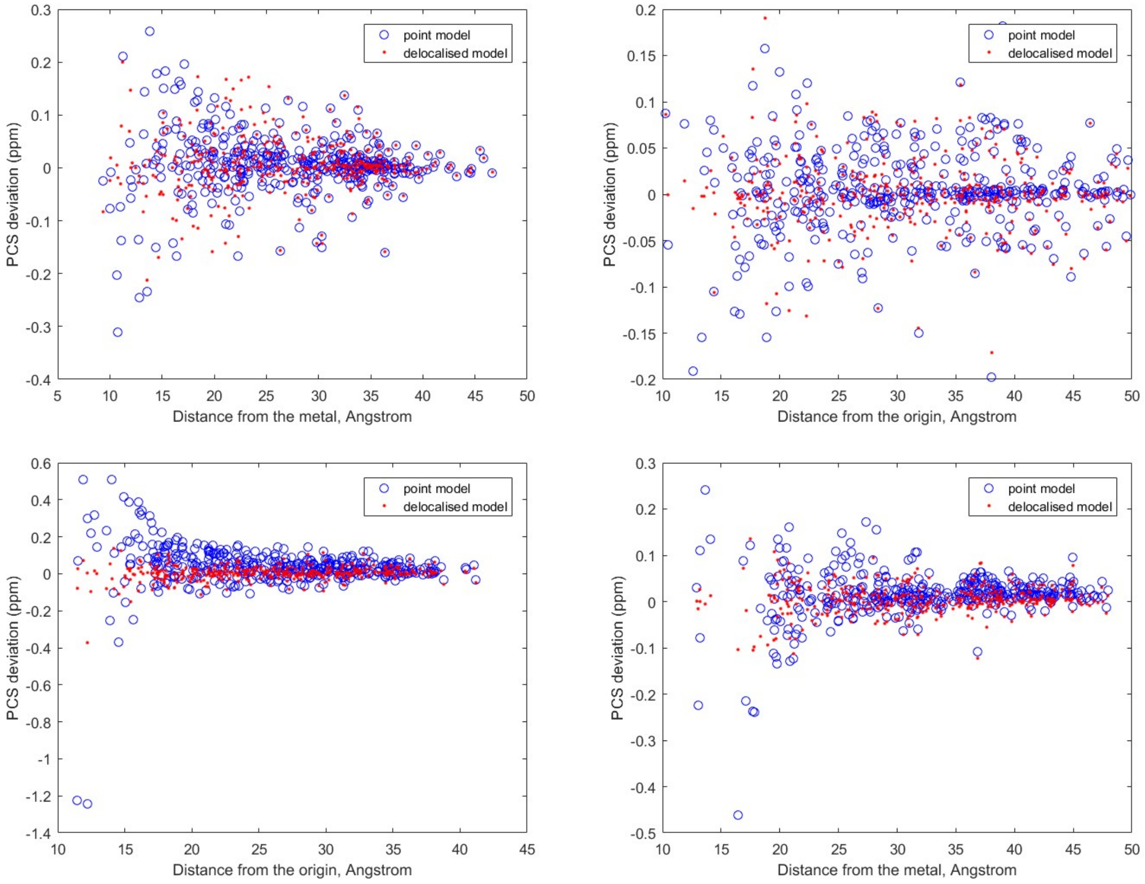

Figure S2. *The difference between the theoretical and the experimental PCS value as a function of the distance from the location of the point paramagnetic tag for the point and the delocalised PCS model, applied to human carbonic anhydrase II with a Tm(III) DOTA-M8 tag attached to the cysteine of S50C mutant (top left), S166C mutant (top right), S217C mutant (bottom left), and S220C mutant (bottom right). Note the significant differences in the Y axis scale between the four plots.*

The residual deviation analysis in Figure S2 indicates that the fitting quality for the S50C and S166C data sets is increased only slightly by allowing for the tag mobility, unlike S217C and S220C, where the delocalised model brings a huge improvement in the fit over the point model when (as per the usual practice in the literature) the proximate nuclei are artificially excluded from the fit.

2. Effective magnetic susceptibility tensor approximation

A significant assumption that is present in both the point model and the delocalised model is that the magnetic susceptibility tensor is the same at every point in the paramagnetic centre distribution. This is clearly an approximation (because the orientation of the tag can vary), but a popular one – made, and occasionally discussed, in hundreds of papers dealing with pseudocontact shift analysis.

We have demonstrated in our previous paper (Ref 21 in the main text) that the general equation for the pseudocontact shift field $\boldsymbol{\sigma}(\mathbf{r})$ produced by a *distributed* magnetic susceptibility tensor $\boldsymbol{\chi}(\mathbf{r})$ is

$$\sigma_{ij}(\mathbf{r})=-\frac{1}{\nabla^2}\sum_k \partial_{ik}\left[\chi_{kj}(\mathbf{r})\rho(\mathbf{r})\right]+\frac{\delta_{ij}}{3}\chi_{ij}(\mathbf{r})\rho(\mathbf{r}) \qquad \text{* MERGEFORMAT (1)}$$

Where the indices run over $\{x,y,z\}$ and $\rho(\mathbf{r})$ is the paramagnetic centre probability density. Introducing the following matrix of second derivative operators

$$\mathbf{D}=\begin{pmatrix}\partial_{xx} & \partial_{xy} & \partial_{xz}\\ \partial_{yx} & \partial_{yy} & \partial_{yz}\\ \partial_{zx} & \partial_{zy} & \partial_{zz}\end{pmatrix} \qquad \text{* MERGEFORMAT (2)}$$

allows us to rewrite Equation * MERGEFORMAT (1) in a more compact form as a matrix product:

$$\sigma_{ij}(\mathbf{r})=-\frac{1}{\nabla^2}\left[\mathbf{D}\cdot\left[\boldsymbol{\chi}(\mathbf{r})\rho(\mathbf{r})\right]\right]_{ij}+\frac{\delta_{ij}}{3}\chi_{ij}(\mathbf{r})\rho(\mathbf{r}) \qquad \text{* MERGEFORMAT (3)}$$

Taking the isotropic part (one third of the trace) of both sides and noting that we are only dealing with the traceless part of the susceptibility tensor, we get:

$$\nabla^2\sigma(\mathbf{r})=-\frac{1}{3}\mathrm{Tr}\left[\mathbf{D}\cdot\left[\boldsymbol{\chi}(\mathbf{r})\rho(\mathbf{r})\right]\right] \qquad \text{* MERGEFORMAT (4)}$$

To obtain an accuracy estimate for the effective magnetic susceptibility tensor approximation, we will now separate, without loss of generality, the "true" coordinate-dependent susceptibility tensor $\boldsymbol{\chi}(\mathbf{r})$ into the "effective" coordinate-independent part $\boldsymbol{\chi}_0$ and the residual $\boldsymbol{\chi}_1(\mathbf{r})$ that depends on the coordinates:

$$\boldsymbol{\chi}(\mathbf{r})=\boldsymbol{\chi}_0+\boldsymbol{\chi}_1(\mathbf{r}) \qquad \text{* MERGEFORMAT (5)}$$

We will also separate, without loss of generality, the "true" paramagnetic centre probability density $\rho(\mathbf{r})$ into the density $\rho_0(\mathbf{r})$ that we have extracted using the effective susceptibility tensor approximation and its difference $\rho_1(\mathbf{r})$ from the true density:

$$\rho(\mathbf{r})=\rho_0(\mathbf{r})+\rho_1(\mathbf{r}) \qquad \text{* MERGEFORMAT (6)}$$

With these substitutions in place, Equation * MERGEFORMAT (4) becomes:

$$\nabla^2\sigma(\mathbf{r})=-\frac{1}{3}\mathrm{Tr}\left[\mathbf{D}\cdot\left[\boldsymbol{\chi}_0\rho_0(\mathbf{r})+\boldsymbol{\chi}_1(\mathbf{r})\rho_0(\mathbf{r})+\boldsymbol{\chi}_0\rho_1(\mathbf{r})+\boldsymbol{\chi}_1(\mathbf{r})\rho_1(\mathbf{r})\right]\right] \text{*}$$

MERGEFORMAT (7)

Our assumption is that we have run the fitting with the "effective" magnetic susceptibility tensor:

$$\nabla^2\sigma(\mathbf{r})\approx-\frac{1}{3}\mathrm{Tr}\left[\mathbf{D}\cdot\left[\chi_0\rho_0(\mathbf{r})\right]\right] \quad \text{* MERGEFORMAT (8)}$$

and obtained the χ_0 matrix and the $\rho_0(\mathbf{r})$ cube. It is also reasonable to assume that we have an estimate χ_{PAF} of the true magnetic susceptibility tensor in the principal axis frame (typically from a DFT calculation), and that it is related by a rotation to the $\chi(\mathbf{r})$ tensor at each point $\mathbf{r}$ in the probability density of the tag:

$$\chi(\mathbf{r})=\mathbf{R}(\mathbf{r})\chi_{\mathrm{PAF}}\mathbf{R}^{-1}(\mathbf{r}) \quad \Rightarrow \quad \chi_1(\mathbf{r})=\mathbf{R}(\mathbf{r})\chi_{\mathrm{PAF}}\mathbf{R}^{-1}(\mathbf{r})-\chi_0 \quad \text{* MERGEFORMAT (9)}$$

After subtracting Equation * MERGEFORMAT (8) from Equation * MERGEFORMAT (7) and removing inconsequential constants, we obtain:

$$0\approx\mathrm{Tr}\left\{\mathbf{D}\cdot\left[\chi_1(\mathbf{r})\rho_0(\mathbf{r})+\chi_0\rho_1(\mathbf{r})+\chi_1(\mathbf{r})\rho_1(\mathbf{r})\right]\right\} \quad \text{* MERGEFORMAT (10)}$$

The function under the square brackets cannot have zero or first order polynomial components by construction – we have a zero boundary condition on the probability density at all cube edges. Therefore, the derivatives can only be close to zero if the function itself is close to the zero matrix; that is also the safest thing to require in order to remove the trace:

$$\chi_1(\mathbf{r})\rho_0(\mathbf{r})+\chi_0\rho_1(\mathbf{r})+\chi_1(\mathbf{r})\rho_1(\mathbf{r})\approx\mathbf{0} \quad \text{* MERGEFORMAT (11)}$$

We can simplify this expression by collecting the terms with a factor of $\rho_1(\mathbf{r})$ and using Equation * MERGEFORMAT (5):

$$\chi_1(\mathbf{r})\rho_0(\mathbf{r})+\chi(\mathbf{r})\rho_1(\mathbf{r})\approx\mathbf{0} \quad \text{* MERGEFORMAT (12)}$$

If the two terms entering this expression are zero individually, everything is fine: that would mean that the two error terms $\rho_1(\mathbf{r})$ and $\chi_1(\mathbf{r})$ are zero. The worst-case scenario is that they are both significant and compensate each other. Because we are building a worst-case estimate, this scenario must be investigated:

$$\chi_1(\mathbf{r})\rho_0(\mathbf{r})\approx-\chi(\mathbf{r})\rho_1(\mathbf{r}) \quad \text{* MERGEFORMAT (13)}$$

After using Equation * MERGEFORMAT (9) for the two susceptibility tensors, we obtain:

$$\left[\chi_0-\mathbf{R}(\mathbf{r})\chi_{\mathrm{PAF}}\mathbf{R}^{-1}(\mathbf{r})\right]\rho_0(\mathbf{r})\approx\mathbf{R}(\mathbf{r})\chi_{\mathrm{PAF}}\mathbf{R}^{-1}(\mathbf{r})\rho_1(\mathbf{r}) \quad \text{* MERGEFORMAT (14)}$$

Taking the 2-norm of both sides and solving for $\rho_1(\mathbf{r})/\rho(\mathbf{r})$, we obtain:

$$\frac{\rho_1(\mathbf{r})}{\rho_0(\mathbf{r})}\approx\frac{\left\|\chi_0-\mathbf{R}(\mathbf{r})\chi_{\mathrm{PAF}}\mathbf{R}^{-1}(\mathbf{r})\right\|}{\left\|\mathbf{R}(\mathbf{r})\chi_{\mathrm{PAF}}\mathbf{R}^{-1}(\mathbf{r})\right\|}=\frac{\left\|\chi_0-\chi(\mathbf{r})\right\|}{\left\|\chi(\mathbf{r})\right\|} \quad \text{* MERGEFORMAT (15)}$$

It follows that the uncertainty resulting from the effective magnetic susceptibility tensor assumption is multiplicative – it would never generate probability density where there was none; it can only scale such density as there is by approximately the factor given in Equation * MERGEFORMAT (15). To use this

equation in practice, the true susceptibility tensor should be estimated using DFT and its rotational range using molecular dynamics. At that point, the right hand side may be evaluated directly. Simple trigonometric estimates for common geometries of tagged proteins indicate that the relative error on the probability density rarely exceeds 20%.

3. DEER traces

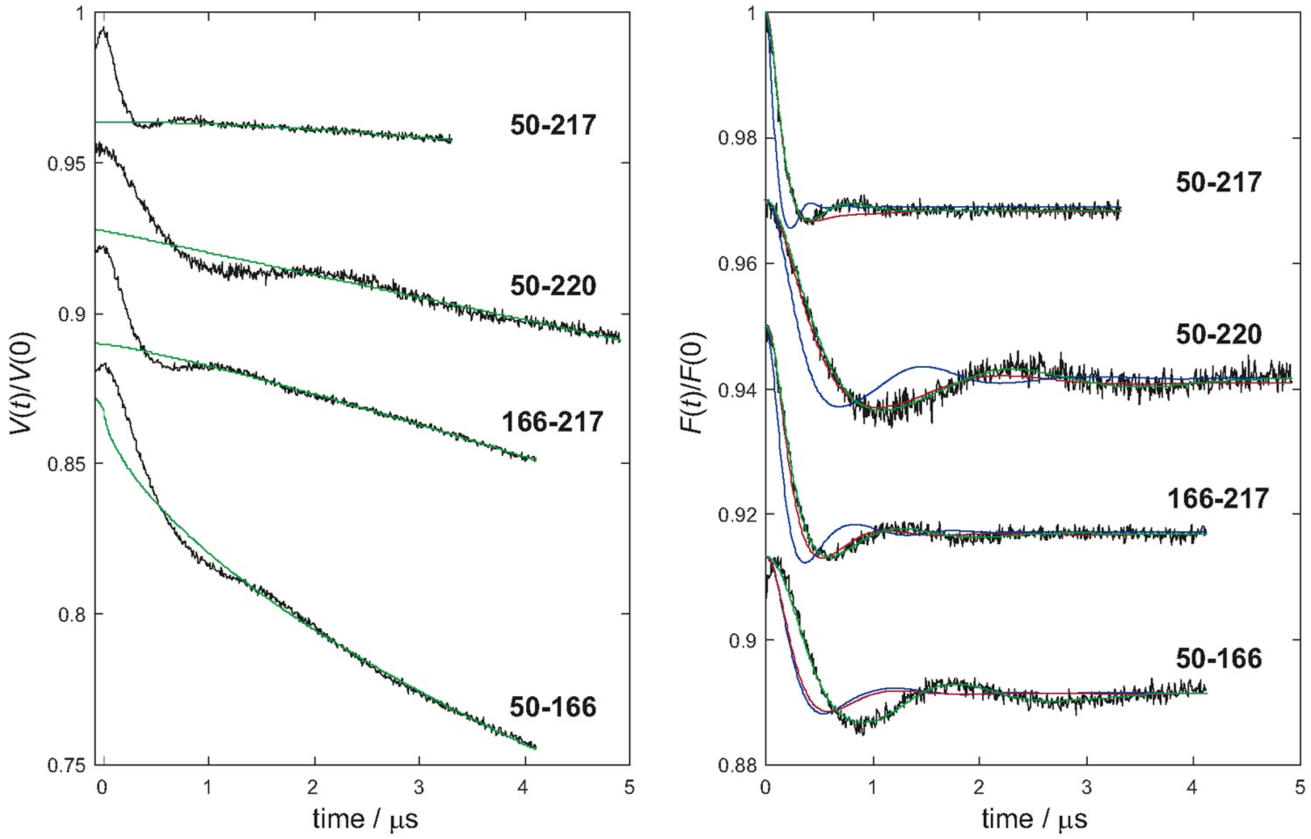

Figure S3. *The left panel shows the experimental Gd-Gd DEER traces (black) and the background fit functions (green). The double mutants are indicated in the figure next to each trace. The right panel shows the background corrected DEER traces (black) and the Tikhonov regularization fits (green). Red lines are simulations obtained using the probability densities reconstructed from single mutant PCS data; blue lines are rotamer library predictions.*

4. Residual anisotropic chemical shifts

Table S3. *PCS and RACS of the residues selected for structure validation calculations. PCS values were determined experimentally from ^{1}H-^{15}N HSQC spectra and the RACS were calculated based on their orientation in the PDB structure 3KS3 using tensor parameters obtained from the data sets where those residues were excluded.*

Mutant	Residue	^{1}H		^{15}N		Residue	^{1}H		^{15}N	
		PCS	RACS	PCS	RACS		PCS	RACS	PCS	RACS
**S50C**	**G25**	0.026	0.000	0.033	-0.014	**G183**	-1.296	-0.001	-1.218	0.023
**S166C**		-0.124	0.000	-0.146	-0.047		0.307	-0.001	0.410	0.039
**S217C**		-0.141	0.001	-0.189	-0.032		-0.079	0.000	-0.068	-0.002
**S220C**		-0.080	-0.001	-0.045	0.031		-0.319	-0.001	-0.334	-0.015
**S50C**	**V31**	0.135	-0.002	0.083	0.025	**T193**	0.591	-0.002	0.646	0.047
**S166C**		-0.096	-0.003	-0.178	0.066		-0.030	0.000	0.025	0.037
**S217C**		-0.592	0.001	-0.538	-0.021		-0.865	0.003	-0.864	-0.004
**S220C**		-0.087	0.000	-0.080	-0.022		-0.006	-0.002	0.015	0.038
**S50C**	**F66**	-0.149	-0.003	-0.079	0.047	**E205**	-0.004	0.001	-0.035	-0.007
**S166C**		-1.196	-0.003	-1.299	0.062		-0.151	0.005	-0.119	-0.039
**S217C**		0.011	0.000	-0.019	-0.009		-0.145	0.002	-0.167	-0.003
**S220C**		-0.447	0.000	-0.395	0.011		-0.083	-0.001	-0.095	0.005
**S50C**	**G132**	-0.243	-0.003	-0.236	0.024	**T208**	0.307	-0.003	0.385	0.048
**S166C**		-0.228	-0.004	-0.297	0.060		-0.137	-0.005	-0.110	0.061
**S217C**		-0.055	0.002	-0.077	-0.026		-0.275	-0.001	-0.329	-0.013
**S220C**		-0.092	0.000	-0.094	-0.019		-0.085	0.000	-0.063	0.013
**S50C**	**Q136**	-0.162	-0.001	-0.089	0.030	**W209**	0.418	0.000	0.469	-0.019
**S166C**		-0.167	-0.004	-0.230	0.062		-0.101	-0.001	-0.186	-0.039
**S217C**		-0.091	0.002	-0.111	-0.027		-0.456	0.004	-0.513	-0.062
**S220C**		-0.078	0.002	-0.092	-0.010		-0.067	0.002	-0.061	-0.004

5. Raw chemical shift data

Residues 1 to 21 could not be assigned. Proline residues 21, 30, 42, 46, 83, 138, 155, 181, 186, 195, 201, 202, 215, 237, 247 and 250 (no resonance in ^{1}H-^{15}N HSQC) are omitted.

Table S4. *Chemical shifts (in ppm), obtained from ^{1}H-^{15}N HSQC, for diamagnetic (Lu) and paramagnetic (Tm) tagged (8S-DOTA-M8) human carbonic anhydrase II, mutants S50C and S166C.*

	**S50C-Lu**		**S50C-Tm**		**S166C-Lu**		**S166C-Tm**	
**Residue**	**HN**	**N**	**HN**	**N**	**HN**	**N**	**HN**	**N**
I22	8.07	120.4	8.06	120.6	8.06	120.6	7.91	120.5
A23	8.67	123.0	8.71	123.0	8.70	123.0	8.54	122.9
K24	7.21	114.8	7.23	114.8	7.21	114.8	7.08	114.7
G25	8.32	108.8	8.34	108.8	8.32	108.8	8.20	108.7
E26	9.37	117.7	9.42	117.8	9.37	117.7	9.27	117.7
R27	8.87	121.4	8.92	121.5	8.86	121.5	8.75	121.4
Q28	7.71	115.5	7.81	115.5	7.71	115.5	7.61	115.3
S29	8.07	117.8	8.16	118.1	8.08	117.9	7.92	117.8
V31	6.30	107.0	6.44	107.1	6.30	106.9	6.21	106.8
D32	8.33	118.3	8.46	118.3	8.35	118.3	8.27	118.2
I33	8.73	128.5	8.90	128.6	8.73	128.5	8.71	128.4
D34	7.53	128.2	7.73	128.4	7.53	128.2	7.50	128.2
T35	10.30	122.3	10.49	122.4	10.30	122.2	10.31	122.2
H36	8.80	118.9	9.01	119.0	8.82	118.8	8.83	118.8
T37	7.65	108.6	7.86	108.8	7.65	108.7	7.65	108.6
A38	7.49	127.6	7.75	127.8	7.48	127.5	7.48	127.5
K39	8.15	122.7	8.51	123.1	8.15	122.7	8.14	122.7
Y40	8.72	126.7	9.13	127.2	8.72	126.8	8.73	126.8
D41	7.76	128.9	8.41	129.4	7.74	128.8	7.74	128.8
S43	8.50	115.2	9.28	116.1	8.50	115.2	8.50	115.2
L44	7.01	123.1	8.05	124.3	7.00	123.1	6.99	123.1
K45	7.58	123.2	9.53	125.2	7.58	123.2	7.56	123.2
L47	8.98	126.4	11.98	130.2	8.97	126.1	8.97	126.1
S48	8.58	121.4	.	.	8.53	120.2	8.49	120.1
V49	8.27	126.2	.	.	8.41	127.6	8.40	127.6
S50	8.52	127.0	.	.	8.38	122.8	8.29	122.6
Y51	8.90	123.5	.	.	9.00	124.5	8.93	124.4
D52	8.74	121.7	.	.	8.90	121.5	8.80	121.3
Q53	8.05	114.4	.	.	8.07	114.4	7.88	114.2
A54	7.40	120.1	3.83	117.2	7.42	120.2	7.16	120.0
T55	9.40	120.8	8.21	119.6	9.44	120.7	8.95	120.1
S56	10.02	126.6	9.28	125.9	10.00	126.4	9.17	125.6
L57	8.94	117.5	8.54	117.2	8.92	117.6	7.70	116.3
R58	7.21	114.8	6.92	114.4	7.20	114.5	5.57	112.6
I59	9.05	122.1	8.84	121.9	9.03	122.0	5.73	119.1
L60	8.72	123.9	8.50	123.6	8.72	123.9	6.49	121.5
N61	8.45	121.2	8.33	121.1	.	.	.	.
N62	8.05	123.3	7.94	123.2	8.05	123.3	6.63	122.0
G63	9.71	108.9	9.63	108.9	9.70	109.1	8.35	107.8
H64	.	.	.	.	.	.	.	.
A65	.	.	.	.	8.62	123.5	7.56	122.6

F66	7.23	110.1	7.08	110.0	7.24	110.1	6.04	108.8
N67	9.00	120.8	8.80	120.6	8.95	120.8	7.37	119.4
V68	8.70	122.0	8.36	121.7	8.68	122.0	7.63	120.8
E69	8.49	124.0	8.16	123.5	8.46	123.9	7.09	122.7
F70	8.65	119.1	8.16	118.6	8.63	119.0	7.92	118.1
D71	8.46	117.0	8.02	116.8	8.44	117.1	7.80	116.5
D72	8.97	131.6	8.63	131.2	8.99	131.7	8.56	131.4
S73	8.76	115.6	8.89	115.7	8.72	115.5	8.37	115.1
Q74	7.66	117.5	8.34	118.3	7.71	117.8	7.42	117.4
D75	8.86	123.0	.	.	8.88	123.1	8.70	123.0
K76	8.01	124.2	.	.	7.96	124.1	7.71	123.9
A77	8.08	122.5	.	.	8.27	123.5	8.01	123.3
V78	8.37	116.7	.	.	8.40	115.9	8.23	115.6
L79	8.98	122.8	.	.	9.03	123.6	8.87	123.5
K80	8.42	119.9	.	.	8.37	120.5	8.30	120.4
G81	9.06	106.6	.	.	9.03	106.5	8.97	106.5
G82	7.13	109.0	11.28	112.8	7.04	108.7	7.02	108.7
L84	7.80	120.3	10.13	122.2	7.78	120.3	7.72	120.3
D85	9.08	125.2	10.01	126.3	9.09	125.2	9.02	125.1
G86	7.92	109.5	9.00	110.3	7.92	109.5	7.84	109.5
T87	8.50	116.4	7.40	115.7	8.53	116.7	8.42	116.6
Y88	8.44	125.8	9.28	125.9	8.44	126.0	8.30	125.9
R89	8.63	123.0	7.60	121.6	8.60	122.8	8.40	122.5
L90	8.55	124.1	6.92	122.8	8.54	123.9	8.28	123.5
I91	.	.	.	.	.	.	.	.
Q92	7.30	115.2	6.89	114.8	7.30	115.1	6.84	114.6
F93	8.63	113.5	8.22	113.1	8.60	113.4	7.89	112.9
H94	8.22	113.1	7.98	112.9	8.21	113.1	7.72	112.6
F95	9.18	117.3	8.99	117.0	9.16	117.2	8.51	116.7
H96	8.74	115.0	8.68	115.0	8.76	115.2	8.45	114.7
W97	9.53	119.1	9.48	119.1	9.54	119.2	9.13	118.9
G98	8.10	108.0	8.04	107.9	8.11	108.0	7.94	107.7
S99	8.41	111.9	8.39	111.8	8.42	111.9	8.18	111.6
L100	7.35	119.0	7.32	118.9	7.36	119.0	7.23	118.9
D101	8.91	120.5	8.88	120.5	8.91	120.5	8.98	120.5
G102	7.69	102.8	7.67	102.7	7.69	102.8	7.73	102.8
Q103	7.87	114.9	7.85	114.9	7.86	114.9	7.85	114.9
G104	8.27	106.3	8.27	106.3	8.27	106.3	8.17	106.1
S105	7.36	107.7	7.35	107.7	7.36	107.7	7.28	107.7
E106	8.31	119.0	8.31	119.1	8.31	119.0	8.18	118.8
H107	10.69	117.8	10.78	117.9	10.69	117.8	10.59	117.7
T108	7.38	109.3	7.49	109.3	7.37	109.3	7.32	109.2
V109	7.64	118.5	7.74	118.6	7.63	118.5	7.63	118.5
D110	9.71	132.3	9.87	132.4	9.72	132.3	9.72	132.3
K111	9.88	109.8	9.99	109.9	9.88	109.8	9.87	109.8
K112	8.26	124.6	8.35	124.6	8.26	124.6	8.27	124.6
K113	7.98	122.0	8.03	122.1	7.98	122.0	7.99	122.1
Y114	8.20	121.4	8.22	121.3	8.19	121.3	8.19	121.4
A115	7.58	120.9	7.57	120.9	7.59	120.9	7.69	121.0
A116	8.16	112.0	8.13	112.0	8.16	112.0	8.20	112.1

E117	9.44	120.9	9.38	120.9	9.41	120.9	9.27	120.8
L118	9.90	130.5	9.83	130.4	9.90	130.5	9.84	130.4
H119	9.00	125.1	8.86	125.2	9.00	125.2	8.71	124.9
L120	9.03	123.7	8.97	123.6	9.03	123.6	8.84	123.5
V121	9.22	126.6	8.93	126.3	9.21	126.6	8.85	126.2
H122	8.55	124.1	8.50	124.0	8.58	124.2	8.34	123.9
W123	9.10	119.1	8.57	118.7	9.10	119.0	8.83	118.8
N124	9.08	120.0	8.76	119.4	9.06	119.8	8.89	119.5
T125	8.21	117.4	6.94	116.2	8.22	117.3	8.06	117.2
K127	7.96	122.9	6.77	121.7	7.96	123.0	7.83	123.0
Y128	7.69	115.6	6.84	114.7	7.69	115.6	7.54	115.5
G129	7.88	107.3	7.13	106.7	7.89	107.4	7.74	107.3
D130	7.45	116.3	7.00	115.8	7.46	116.3	7.30	116.1
F131	7.63	119.6	7.28	119.3	7.63	119.6	7.38	119.4
G132	8.11	104.5	7.87	104.3	8.11	104.5	7.88	104.2
K133	7.65	119.6	7.39	119.3	7.65	119.6	7.47	119.3
A134	7.95	123.4	7.64	123.1	7.95	123.4	7.77	123.3
V135	7.94	113.4	7.73	113.1	7.94	113.4	7.74	113.2
Q136	6.59	113.5	6.43	113.4	6.59	113.5	6.42	113.3
Q137	7.68	119.1	7.54	119.0	7.68	119.1	7.53	118.9
D139	7.92	114.6	7.86	114.6	7.93	114.6	7.82	114.5
G140	8.28	108.1	8.24	108.1	8.29	108.1	8.15	107.9
L141	8.76	115.6	8.79	115.6	8.75	115.6	8.59	115.4
A142	8.90	123.5	9.03	123.8	8.91	123.5	8.72	123.4
V143	7.28	124.2	7.68	124.6	7.28	124.2	7.13	124.0
L144	8.48	128.4	8.66	128.6	8.46	128.2	8.29	128.1
G145	9.96	114.4	10.26	114.5	9.93	114.2	9.85	114.1
I146	9.14	124.2	9.17	124.3	9.16	124.3	9.10	124.3
F147	10.13	129.0	10.19	128.9	10.13	129.0	10.20	129.0
L148	8.24	120.3	8.22	120.3	8.24	120.4	8.35	120.4
K149	8.98	119.3	8.92	119.3	8.98	119.4	9.30	119.6
V150	8.48	121.4	8.43	121.4	8.47	121.5	8.79	121.7
G151	9.13	118.2	9.02	118.1	9.12	118.1	9.75	118.8
S152	8.37	124.6	8.25	124.5	8.38	124.5	9.06	125.2
A153	8.39	121.1	8.21	120.9	8.40	121.3	9.61	122.4
K154	8.72	123.9	8.48	123.6	8.72	123.9	10.07	125.2
G156	.	.	.	.	.	.	.	.
L157	7.56	116.8	7.13	116.3	7.55	116.8	9.03	118.3
Q158	7.80	122.4	7.40	122.0	7.82	122.3	10.24	124.9
K159	8.85	115.8	8.42	115.5	8.85	115.8	11.96	119.5
V160	7.04	113.2	6.66	112.8	7.07	113.7	8.73	115.5
V161	7.30	114.5	7.01	114.1	7.30	114.7	.	.
D162	8.40	117.5	8.14	117.3	8.42	117.6	.	.
V163	7.15	113.9	6.91	113.7	7.08	114.6	.	.
L164	7.08	121.7	6.88	121.5	7.10	121.1	.	.
D165	8.23	116.2	8.09	116.1	8.15	116.2	.	.
S166	8.01	113.5	7.87	113.4	.	.	.	.
I167	7.43	116.4	7.29	116.3	8.14	114.5	-3.06	104.1
K168	7.41	117.5	7.31	117.4	7.71	117.8	0.28	111.8
T169	6.59	131.5	6.51	131.4	6.58	131.4	2.74	127.9

K170	7.49	120.5	7.42	120.5	7.49	120.6	5.77	118.8
G171	8.91	116.9	8.81	116.9	8.92	116.9	7.24	115.5
K172	7.81	120.2	7.73	120.2	7.81	120.2	5.82	118.4
S173	8.36	113.7	8.27	113.6	8.38	113.8	.	.
A174	9.03	123.3	8.89	123.2	9.00	122.8	.	.
D175	8.63	122.1	8.51	121.9	8.61	122.2	.	.
F176	8.03	125.4	7.80	125.0	8.00	125.0	.	.
T177	8.11	114.8	7.83	114.5	8.07	114.7	.	.
N178	9.72	115.5	9.24	114.9	9.74	115.6	8.46	114.3
F179	8.28	118.8	7.58	118.1	8.29	118.9	7.41	118.3
D180	8.04	128.0	7.01	126.8	8.00	128.1	8.14	128.0
R182	8.33	118.3	6.34	116.5	8.35	118.3	8.43	118.4
G183	7.31	102.7	6.01	101.5	7.31	102.7	7.62	103.1
L184	7.24	117.3	6.34	116.5	7.26	117.3	7.54	117.7
L185	6.64	113.2	5.88	112.6	6.65	113.1	6.93	113.3
E187	8.18	116.3	8.13	116.3	8.19	116.4	8.45	116.6
S188	7.64	111.3	7.76	111.5	7.64	111.3	7.83	111.5
L189	8.65	125.7	9.12	126.1	8.65	125.7	8.75	125.8
D190	7.43	121.9	7.98	122.5	7.42	121.9	7.43	121.9
Y191	8.77	119.0	9.60	119.7	8.77	118.9	8.79	118.9
W192	9.55	117.2	10.11	117.8	9.55	117.2	9.56	117.1
T193	9.71	115.2	10.30	115.8	9.71	115.2	9.68	115.2
Y194	8.05	125.5	8.55	126.1	8.06	125.6	7.99	125.5
G196	9.38	110.8	9.60	111.0	9.37	110.8	9.26	110.6
S197	8.70	120.0	8.82	120.3	8.70	120.0	8.55	120.0
L198	8.03	118.3	8.02	118.3	8.02	118.3	7.82	118.1
T199	.	.	.	.	.	.	.	.
T200	6.98	108.8	6.98	108.6	7.00	108.8	6.70	108.4
L203	9.12	120.2	9.13	120.3	9.14	120.3	8.88	120.0
L204	5.95	111.4	5.92	111.3	5.96	111.3	5.74	111.1
E205	8.68	125.0	8.68	125.0	8.68	125.0	8.53	124.9
C206	7.46	113.7	7.49	113.8	7.47	113.9	7.31	113.6
V207	7.25	116.7	7.36	116.8	7.25	116.7	7.10	116.5
T208	8.35	125.3	8.65	125.7	8.35	125.4	8.21	125.3
W209	8.24	129.3	8.65	129.8	8.23	129.3	8.13	129.2
I210	8.78	127.2	9.31	127.9	8.77	127.3	8.67	127.3
V211	9.75	128.4	10.29	128.9	9.75	128.4	9.71	128.4
L212	8.95	126.5	9.28	127.0	8.98	126.7	8.98	126.8
K213	7.41	122.4	7.83	122.7	7.41	122.3	7.47	122.4
E214	8.90	121.6	9.16	121.8	8.90	121.6	9.02	121.7
I216	8.93	113.4	8.87	113.3	8.93	113.4	9.18	113.6
S217	8.10	116.7	7.94	116.5	8.10	116.6	8.62	117.1
V218	8.55	115.8	8.42	115.7	8.54	115.9	9.13	116.5
S219	8.42	115.9	8.24	115.8	8.42	116.0	9.56	117.1
S220	9.32	117.3	9.21	117.2	9.31	117.3	10.16	118.2
E221	8.41	118.4	8.29	118.3	8.41	118.5	9.60	119.6
Q222	7.55	117.9	7.39	117.8	7.54	118.0	8.89	119.5
V223	7.38	114.7	7.24	114.6	7.35	114.6	8.11	115.3
L224	8.03	120.9	7.91	120.8	8.01	120.9	8.58	121.3
K225	6.92	115.0	6.79	114.9	6.92	115.1	7.44	115.5

F226	7.13	118.7	7.00	118.6	7.13	118.7	6.99	118.4
R227	6.70	109.1	6.60	108.9	6.71	109.0	6.24	108.4
K228	6.86	114.1	6.76	114.0	6.87	114.1	5.78	112.9
L229	7.11	119.3	7.01	119.3	7.10	119.2	5.45	117.3
N230	8.92	118.4	8.84	118.4	8.89	118.3	6.49	116.2
F231	8.89	117.2	8.83	117.1	8.90	117.4	7.67	116.0
N232	8.17	110.6	8.12	110.6	8.17	110.7	6.92	109.5
G233	8.68	104.8	8.64	104.7	8.68	104.8	7.70	103.6
E234	8.40	121.3	8.36	121.2	8.40	121.3	7.11	120.0
G235	9.09	114.1	9.05	114.1	9.09	114.1	7.86	113.0
E236	7.35	120.0	7.32	119.9	7.36	120.0	6.37	119.0
E238	8.27	123.3	8.24	123.2	8.27	123.3	7.34	122.3
E239	9.03	130.0	8.98	130.0	9.01	129.9	7.90	128.9
L240	8.75	126.0	8.71	125.9	8.75	126.0	7.90	125.0
M241	8.63	123.0	8.56	123.0	8.66	123.1	7.51	121.9
V242	6.74	114.8	6.69	114.8	6.75	114.8	6.04	114.1
D243	7.16	114.8	7.10	114.7	7.14	114.8	6.73	114.3
N244	8.40	119.3	8.36	119.2	8.40	119.3	7.95	118.8
W245	6.50	114.2	6.47	114.1	6.51	114.2	6.16	113.8
R246	10.44	127.0	10.42	126.9	10.40	126.8	10.13	126.5
A248	8.02	120.3	8.07	120.3	8.01	120.4	7.89	120.2
Q249	8.70	122.0	8.81	122.1	8.71	122.0	8.61	121.8
L251	8.70	125.9	8.81	126.0	8.70	125.8	8.63	125.8
K252	8.89	114.6	8.99	114.6	8.89	114.6	8.83	114.5
N253	8.70	119.7	8.82	119.8	8.71	119.7	8.65	119.7
R254	7.31	118.5	7.47	118.7	7.32	118.6	7.26	118.5
Q255	8.60	118.2	8.85	118.5	8.59	118.2	8.55	118.3
I256	8.94	124.4	9.22	124.8	8.93	124.4	8.90	124.4
K257	8.81	126.6	9.28	127.0	8.81	126.6	8.77	126.7
A258	8.40	123.6	8.93	124.2	8.40	123.6	8.39	123.6
S259	8.98	116.5	9.74	117.2	8.97	116.4	8.98	116.4
F260	6.67	118.0	7.32	118.7	6.67	118.0	6.69	118.0
K261	7.77	124.4	8.31	125.0	7.76	124.4	7.81	124.4

Table S5. *Chemical shifts (in ppm), obtained from ^{1}H-^{15}N HSQC, for diamagnetic (Lu) and paramagnetic (Tm) tagged (8S-DOTA-M8) human carbonic anhydrase II, mutants S217C and S220C.*

	**S217C-Lu**		**S217C-Tm**		**S220C-Lu**		**S220C-Tm**	
**Residue**	**HN**	**N**	**HN**	**N**	**HN**	**N**	**HN**	**N**
I22	8.06	120.6	7.98	120.4	8.07	120.6	7.98	120.5
A23	8.70	123.0	8.60	122.9	8.70	123.0	8.60	122.9
K24	7.21	114.8	7.11	114.7	7.21	114.7	.	.
G25	8.32	108.8	8.18	108.6	8.32	108.8	8.24	108.8
E26	9.37	117.7	9.20	117.5	9.36	117.7	9.31	117.6
R27	8.87	121.4	8.65	121.2	8.87	121.5	8.80	121.5
Q28	7.72	115.5	7.46	115.1	7.71	115.4	7.64	115.4
S29	8.08	117.9	7.81	117.6	8.09	117.9	7.98	117.8
V31	6.30	107.0	5.71	106.4	6.30	106.9	6.21	106.9

D32	8.35	118.3	7.73	117.6	8.33	118.2	8.30	118.2
I33	8.73	128.5	7.54	127.3	8.72	128.5	8.75	128.5
D34	7.52	128.2	6.43	127.0	7.52	128.2	7.57	128.3
T35	10.30	122.2	8.54	120.6	10.30	122.2	10.43	122.3
H36	8.82	118.8	7.44	117.4	8.81	118.8	8.93	119.0
T37	7.66	108.6	6.57	107.6	7.65	108.6	7.73	108.7
A38	7.49	127.5	6.34	126.5	7.49	127.5	7.56	127.6
K39	8.15	122.7	7.34	121.9	8.14	122.7	8.17	122.7
Y40	8.72	126.8	7.92	126.0	8.72	126.7	8.77	126.8
D41	7.75	128.9	7.18	128.3	7.73	128.8	7.75	128.8
S43	8.50	115.2	8.25	115.1	8.50	115.2	8.51	115.2
L44	7.01	123.1	6.76	122.9	6.99	123.1	6.99	123.1
K45	7.58	123.1	7.45	123.0	7.58	123.2	7.56	123.2
L47	8.98	126.0	9.05	126.1	8.97	126.1	8.94	126.0
S48	8.52	120.1	8.59	120.2	8.54	120.2	8.47	120.2
V49	8.41	127.6	8.70	127.8	8.41	127.6	8.33	127.5
S50	8.38	122.8	8.51	122.9	8.38	122.8	8.28	122.7
Y51	9.02	124.6	9.23	124.7	9.00	124.6	8.87	124.4
D52	8.89	121.4	9.10	121.5	8.89	121.3	8.76	121.2
Q53	8.06	114.4	8.19	114.4	8.05	114.4	7.93	114.3
A54	7.42	120.2	7.48	120.2	7.43	120.2	7.28	120.0
T55	9.43	120.7	9.43	120.7	9.44	120.7	9.29	120.5
S56	10.01	126.5	9.97	126.5	10.01	126.5	9.82	126.3
L57	8.94	117.6	8.89	117.6	8.94	117.6	8.75	117.4
R58	7.21	114.8	7.15	114.7	7.21	114.7	7.01	114.5
I59	9.06	122.1	9.02	122.0	9.05	122.0	8.81	121.8
L60	8.70	123.8	8.68	123.7	8.70	123.7	8.41	123.5
N61	8.45	121.1	.	.	8.45	121.1	8.21	120.9
N62	8.04	123.3	8.04	123.3	8.05	123.3	7.78	123.0
G63	9.71	109.1	9.74	109.1	9.71	109.1	9.46	108.8
H64	.	.	.	.	.	.	.	.
A65	8.61	123.6	.	.	8.63	123.5	.	.
F66	7.22	110.1	7.23	110.1	7.23	110.1	6.78	109.7
N67	8.97	120.8	8.96	120.8	8.97	120.8	8.66	120.5
V68	8.72	122.0	.	.	8.71	122.0	8.30	121.6
E69	8.48	123.9	8.42	123.8	8.48	123.9	8.24	123.7
F70	8.64	119.0	8.58	118.9	8.64	119.0	8.46	118.8
D71	8.44	117.0	8.40	117.0	8.46	117.1	8.32	117.0
D72	8.99	131.8	8.97	131.8	8.99	131.8	8.87	131.7
S73	8.73	115.5	8.72	115.4	8.72	115.5	8.62	115.3
Q74	7.70	117.7	7.70	117.8	7.70	117.7	7.61	117.6
D75	8.88	123.1	8.86	123.1	8.89	123.1	8.73	123.0
K76	7.96	124.1	7.98	124.2	7.96	124.2	7.86	124.0
A77	8.28	123.5	8.29	123.5	8.29	123.5	8.17	123.4
V78	8.39	115.9	8.45	115.9	8.40	115.9	8.29	115.7
L79	9.02	123.6	9.00	123.5	9.03	123.6	8.92	123.5
K80	8.38	120.6	8.39	120.5	8.37	120.5	8.29	120.4
G81	9.03	106.5	9.02	106.5	9.03	106.5	8.98	106.4
G82	7.06	108.7	7.02	108.7	7.03	108.8	7.00	108.7
L84	7.78	120.3	7.61	120.1	7.79	120.3	7.74	120.3

D85	9.09	125.2	8.96	125.2	9.09	125.2	9.05	125.2
G86	7.92	109.5	7.87	109.5	7.92	109.5	7.87	109.5
T87	8.53	116.7	8.50	116.7	8.53	116.7	8.47	116.6
Y88	8.45	126.0	8.41	126.0	8.44	126.0	8.36	125.9
R89	8.60	122.8	8.54	122.7	8.60	122.7	8.50	122.6
L90	8.54	123.8	8.49	123.9	8.55	123.8	8.42	123.6
I91	.	.	.	.	.	.	.	.
Q92	7.30	115.1	7.19	114.9	7.30	115.1	7.10	114.9
F93	8.61	113.5	8.52	113.3	8.59	113.4	8.32	113.2
H94	8.20	113.1	8.03	113.0	8.21	113.0	7.88	112.7
F95	9.15	117.2	9.12	117.1	9.18	117.2	8.71	116.8
H96	8.74	115.1	8.72	115.1	8.81	115.2	8.31	114.7
W97	9.55	119.2	9.75	119.4	9.51	119.1	8.79	118.3
G98	8.14	108.0	8.77	108.6	8.03	107.8	6.78	106.6
S99	8.43	112.0	9.01	112.5	8.47	112.1	7.57	111.3
L100	7.39	119.0	8.17	119.8	7.26	119.6	6.48	118.9
D101	8.93	120.6	10.34	122.0	8.74	120.4	.	.
G102	7.68	102.8	9.19	104.3	7.81	103.6	6.37	102.6
Q103	7.86	114.9	8.97	115.9	7.79	114.8	.	.
G104	8.26	106.3	8.71	106.8	8.28	106.6	7.73	106.0
S105	7.36	107.7	7.72	108.0	7.36	107.8	7.00	107.4
E106	8.31	119.0	8.00	118.8	8.34	119.0	8.10	118.7
H107	10.71	117.8	10.14	117.2	10.67	117.8	10.52	117.7
T108	7.37	109.3	6.50	108.4	7.38	109.3	7.35	109.2
V109	7.64	118.6	6.23	117.0	7.69	118.6	7.83	118.7
D110	9.72	132.3	8.07	130.6	9.72	132.3	9.85	132.5
K111	9.88	109.8	8.84	108.8	9.89	109.8	9.99	109.9
K112	8.26	124.5	7.04	123.6	8.25	124.5	8.44	124.7
K113	7.98	121.6	8.14	121.6	7.97	122.1	8.13	122.3
Y114	8.15	121.1	.	.	8.20	121.2	8.20	121.2
A115	7.65	120.8	.	.	7.71	121.0	7.26	122.0
A116	8.11	111.7	8.96	112.5	8.15	112.0	7.91	111.6
E117	9.41	120.9	9.43	120.9	9.45	120.8	8.98	120.5
L118	9.92	130.5	9.33	130.1	9.92	130.5	9.63	130.2
H119	9.00	125.2	8.72	124.8	9.00	125.1	8.67	124.9
L120	9.02	123.6	8.63	123.3	9.03	123.6	8.83	123.4
V121	9.21	126.6	9.03	126.3	9.23	126.6	9.02	126.4
H122	8.58	124.2	8.40	124.0	8.59	124.2	8.45	124.1
W123	9.10	119.0	9.00	118.9	9.10	119.0	8.99	118.9
N124	9.06	119.8	8.95	119.8	9.07	119.8	8.98	119.7
T125	8.22	117.3	8.16	117.2	8.22	117.3	8.14	117.2
K127	7.96	123.0	7.91	123.0	7.96	123.0	7.90	122.9
Y128	7.68	115.5	7.63	115.5	7.69	115.5	7.62	115.4
G129	7.89	107.4	7.85	107.3	7.89	107.4	7.82	107.4
D130	7.47	116.3	7.42	116.3	7.47	116.3	7.39	116.3
F131	7.63	119.6	7.58	119.5	7.65	119.6	7.56	119.5
G132	8.11	104.5	8.06	104.4	8.11	104.5	8.02	104.4
K133	7.65	119.6	7.59	119.5	7.65	119.6	7.57	119.5
A134	7.96	123.4	7.89	123.3	7.96	123.5	7.88	123.4
V135	7.94	113.4	7.85	113.3	7.95	113.4	7.86	113.3

Q136	6.59	113.5	6.50	113.4	6.58	113.5	6.50	113.5
Q137	7.68	119.1	7.57	118.9	7.68	119.1	7.60	119.1
D139	7.93	114.6	7.79	114.4	7.94	114.6	7.88	114.5
G140	8.29	108.1	8.14	108.0	8.28	108.1	8.21	108.0
L141	8.75	115.6	8.58	115.4	8.77	115.6	8.69	115.6
A142	8.91	123.6	8.72	123.3	8.90	123.5	8.80	123.4
V143	7.28	124.2	6.93	123.8	7.29	124.2	7.18	124.1
L144	8.46	128.1	8.08	127.7	8.47	128.1	8.32	128.0
G145	9.92	114.2	9.09	113.4	9.93	114.2	9.82	114.1
I146	9.14	124.3	8.25	123.2	9.17	124.3	8.98	124.1
F147	10.10	129.0	7.96	127.1	10.15	129.0	10.11	129.0
L148	8.20	120.5	10.16	122.6	8.22	120.1	8.11	120.1
K149	9.08	121.9	.	.	8.93	118.6	.	.
V150	8.27	120.3	.	.	8.62	121.4	9.27	122.3
G151	7.64	120.2	.	.	9.14	118.3	.	.
S152	8.34	122.3	.	.	8.37	124.5	.	.
A153	.	.	.	.	8.38	121.0	7.42	120.2
K154	8.70	124.1	.	.	8.67	123.9	.	.
G156	.	.	.	.	.	.	.	.
L157	7.53	116.7	.	.	7.58	116.8	6.66	116.0
Q158	7.79	122.2	7.43	121.9	7.80	122.3	6.83	121.4
K159	8.81	115.8	8.53	115.5	8.83	115.8	8.15	115.2
V160	7.03	113.2	.	.	7.04	113.1	6.31	112.5
V161	7.30	114.3	7.23	114.3	7.31	114.1	6.32	113.2
D162	8.40	117.6	8.26	117.5	8.40	117.8	7.55	117.0
V163	7.14	113.8	7.06	113.7	7.15	113.8	6.54	113.3
L164	7.07	121.6	7.06	121.6	7.08	121.7	6.44	121.1
D165	8.22	116.2	8.21	116.2	8.24	116.2	7.76	115.8
S166	8.00	113.5	7.98	113.5	8.00	113.6	7.69	113.4
I167	7.42	116.4	7.46	116.3	7.42	116.4	7.12	116.1
K168	7.42	117.4	7.44	117.4	7.40	117.5	7.20	117.3
T169	6.60	131.6	6.63	131.6	6.59	131.4	6.43	131.2
K170	7.50	120.6	7.53	120.7	7.49	120.5	7.34	120.4
G171	8.89	116.9	8.90	116.9	8.89	116.9	8.71	116.8
K172	7.82	120.2	7.81	120.2	7.80	120.2	7.64	120.0
S173	8.36	113.7	8.35	113.7	8.36	113.8	8.21	113.6
A174	9.03	123.4	9.01	123.3	9.03	123.4	8.82	123.3
D175	8.63	122.0	8.59	122.0	8.63	122.1	8.47	121.9
F176	8.00	125.3	7.95	125.2	8.01	125.3	7.81	125.0
T177	8.12	114.9	8.04	114.8	8.12	114.9	7.92	114.7
N178	9.73	115.5	9.68	115.4	9.73	115.5	9.55	115.2
F179	8.28	118.9	8.21	118.8	8.28	118.9	8.05	118.6
D180	8.02	128.1	7.91	128.0	8.01	128.1	7.71	127.8
R182	8.35	118.3	8.37	118.3	8.36	118.4	8.11	118.2
G183	7.33	102.8	7.25	102.7	7.32	102.6	7.00	102.3
L184	7.26	117.4	7.09	117.2	7.25	117.3	6.91	116.9
L185	6.64	113.1	6.48	112.8	6.65	113.1	6.36	112.8
E187	8.18	116.3	.	.	8.19	116.4	8.18	116.4
S188	7.62	111.6	.	.	7.65	111.4	7.63	111.3
L189	8.63	125.8	9.56	126.4	8.65	125.7	8.63	125.7

D190	7.47	122.0	7.18	121.5	7.42	121.9	7.44	121.8
Y191	8.75	118.8	7.70	117.8	8.76	119.2	.	.
W192	9.56	117.2	8.00	115.6	9.55	117.2	9.55	117.2
T193	9.71	115.1	8.84	114.3	9.71	115.2	9.71	115.2
Y194	8.06	125.5	7.46	124.9	8.06	125.6	8.02	125.6
G196	9.37	110.8	9.04	110.5	9.39	110.8	9.32	110.7
S197	8.70	120.0	8.40	119.8	8.70	120.0	8.62	120.0
L198	8.02	118.3	7.83	118.1	8.02	118.3	7.90	118.2
T199	.	.	.	.	.	.	.	.
T200	7.00	108.8	6.86	108.8	7.00	108.8	6.82	108.6
L203	9.14	120.3	9.03	120.1	9.14	120.3	9.00	120.2
L204	5.96	111.3	5.83	111.1	5.95	111.4	5.84	111.3
E205	8.68	125.0	8.54	124.8	8.68	125.0	8.60	124.9
C206	7.47	113.8	7.28	113.6	7.47	113.8	7.38	113.7
V207	7.25	116.7	7.01	116.5	7.25	116.7	7.17	116.6
T208	8.35	125.4	8.07	125.1	8.35	125.4	8.27	125.3
W209	8.23	129.3	7.77	128.8	8.24	129.4	8.18	129.3
I210	8.77	127.3	8.18	126.7	8.78	127.3	8.69	127.3
V211	9.74	128.4	8.68	127.3	9.75	128.5	9.71	128.4
L212	8.96	126.7	7.30	125.0	8.99	126.7	8.94	126.7
K213	7.40	122.2	.	.	7.41	122.3	7.45	122.4
E214	8.95	122.3	.	.	8.90	121.7	8.97	121.7
I216	8.77	110.2	.	.	8.94	113.3	8.92	113.3
S217	7.72	121.0	.	.	8.07	116.4	.	.
V218	8.59	115.1	.	.	8.56	115.6	.	.
S219	8.38	116.3	.	.	.	.	.	.
S220	9.42	118.1	10.79	119.3	.	.	.	.
E221	8.44	118.8	8.98	119.4	8.48	118.1	.	.
Q222	7.58	118.1	8.08	118.5	7.47	117.4	.	.
V223	7.30	114.3	8.11	114.9	7.47	115.6	.	.
L224	7.98	120.6	8.63	121.2	8.13	120.0	.	.
K225	6.92	115.2	7.27	115.6	6.99	115.7	.	.
F226	7.14	118.7	7.49	119.0	7.12	118.8	.	.
R227	6.70	109.0	7.07	109.3	6.68	108.9	.	.
K228	6.87	114.1	7.12	114.4	6.87	114.4	.	.
L229	7.11	119.2	7.29	119.4	7.11	119.3	.	.
N230	8.92	118.4	9.02	118.6	8.90	118.4	8.62	118.1
F231	8.89	117.3	9.00	117.4	8.89	117.2	8.66	116.9
N232	8.18	110.7	8.26	110.7	8.18	110.7	8.03	110.5
G233	8.68	104.8	8.72	104.8	8.69	104.8	8.62	104.7
E234	8.41	121.3	8.43	121.4	8.41	121.4	8.36	121.3
G235	9.10	114.1	9.13	114.1	9.09	114.1	9.12	114.2
E236	7.35	120.0	7.40	120.1	7.36	120.0	7.38	120.0
E238	8.27	123.3	8.37	123.4	8.27	123.3	8.39	123.4
E239	9.02	130.0	9.13	130.1	9.04	129.9	8.99	129.9
L240	8.75	125.9	8.92	126.2	8.76	125.9	8.61	125.7
M241	8.64	123.0	8.81	123.1	8.65	123.1	8.08	122.6
V242	6.75	114.9	6.96	115.2	6.73	115.0	6.11	114.3
D243	7.11	114.8	7.46	115.1	7.14	114.7	6.27	113.9
N244	8.40	119.3	8.59	119.4	8.38	119.2	7.72	118.5

W245	6.51	114.2	6.61	114.3	6.50	114.2	6.01	113.7
R246	10.39	126.8	10.30	126.8	10.38	126.7	10.08	126.3
A248	8.01	120.4	7.81	120.2	8.01	120.4	7.85	120.2
Q249	8.67	122.0	8.39	121.7	8.71	122.0	8.61	121.9
L251	8.70	125.8	8.32	125.5	8.70	125.8	8.66	125.7
K252	8.90	114.6	8.57	114.3	8.89	114.6	8.87	114.5
N253	8.71	119.7	8.41	119.3	8.71	119.7	8.68	119.6
R254	7.32	118.6	6.96	118.3	7.31	118.6	7.29	118.5
Q255	8.59	118.2	8.16	117.8	8.60	118.3	8.59	118.2
I256	8.93	124.4	8.26	123.7	8.93	124.4	8.94	124.4
K257	8.80	126.6	8.07	125.8	8.81	126.6	8.81	126.6
A258	8.40	123.6	7.57	122.7	8.39	123.6	8.42	123.6
S259	8.97	116.5	8.00	115.6	8.97	116.4	8.99	116.4
F260	6.67	118.0	5.69	117.2	6.67	118.0	6.72	118.1
K261	7.76	124.4	7.13	123.6	7.77	124.4	7.83	124.5

6. Pseudocontact shift data

Residues 1 to 21 could not be assigned. Proline residues 21, 30, 42, 46, 83, 138, 155, 181, 186, 195, 201, 202, 215, 237, 247 and 250 (no resonance in ^{1}H-^{15}N HSQC) are omitted.

Table S6. *PCS (in ppm) for all mutants computed by subtracting the diamagnetic reference (Lu) chemical shifts from the chemical shifts measured for the paramagnetic (Tm)-tagged molecules.*

	S50C		S166C		S217C		S220C	
**Residue**	**HN**	**N**	**HN**	**N**	**HN**	**N**	**HN**	**N**
I22	−0.010*	0.167*	−0.150*	−0.085*	−0.089	−0.136	−0.082	−0.051
A23	0.032*	0.052*	−0.163	−0.094	−0.100	−0.137	−0.094	−0.093
K24	0.019	0.063	−0.132	−0.061	−0.096	−0.098	.	.
G25	0.026	0.033	−0.124	−0.146	−0.141	−0.189	−0.080	−0.045
E26	0.053	0.101	−0.102	−0.034	−0.171	−0.205	−0.057	−0.069
R27	0.055	0.090	−0.111	−0.066	−0.220	−0.220	−0.068	−0.038
Q28	0.094	0.060	−0.102	−0.167	−0.252	−0.361	−0.076	−0.057
S29	0.094	0.235*	−0.160	−0.098	−0.270	−0.303	−0.111	−0.102
V31	0.135	0.083	−0.096	−0.178	−0.592	−0.538	−0.087	−0.080
D32	0.128	−0.006*	−0.072	−0.112	−0.616	−0.660	−0.038	−0.061
I33	0.165	0.131	−0.022	−0.058	−1.192	−1.165	0.033	0.034
D34	0.200	0.166	−0.020	0.010	−1.099	−1.239	0.051	0.034
T35	0.189	0.143	0.014	−0.021	−1.752	−1.612	0.136	0.118
H36	0.207*	0.126*	0.013	0.000	−1.380	−1.384	0.117	0.148
T37	0.214	0.242	0.000	−0.045	−1.087	−1.054	0.080	0.082
A38	0.261	0.206	0.001	−0.003	−1.146	−1.017	0.070	0.020
K39	0.360	0.376	−0.011	0.024	−0.817	−0.857	0.036	0.048
Y40	0.410	0.493	0.005	0.005	−0.800	−0.782	0.051	0.026
D41	0.653	0.571	0.002	−0.037	−0.571	−0.585	0.023	0.029
S43	0.779	0.879	−0.005	−0.042	−0.249	−0.160	0.005	−0.005
L44	1.039	1.213	−0.010	0.024	−0.252	−0.237	−0.001	0.003
K45	1.949	1.928	−0.020	−0.026	−0.130	−0.114	−0.019	−0.022
L47	3.001*	3.747*	0.000	0.051	0.066	0.062	−0.035	−0.044
S48	.	.	−0.037	−0.082	0.072	0.105	−0.063	−0.071
V49	.	.	−0.015	0.026	0.284	0.207	−0.088	−0.077
S50	.	.	−0.088	−0.134	0.124	0.145	−0.107	−0.111
Y51	.	.	−0.068	−0.079	0.216	0.150	−0.130	−0.138
D52	.	.	−0.093	−0.191	0.204	0.116	−0.124	−0.111
Q53	.	.	−0.181	−0.209	0.129	0.061	−0.121	−0.083
A54	−3.566*	−2.908*	−0.254	−0.227	0.066	0.031	−0.145	−0.122
T55	−1.195	−1.220	−0.493	−0.596	0.004	−0.057	−0.151	−0.186
S56	−0.746	−0.698	−0.824	−0.885	−0.034	−0.043	−0.189	−0.196
L57	−0.396	−0.341	−1.216	−1.318	−0.047	−0.091	−0.196	−0.172
R58	−0.296	−0.319	−1.623*	−1.880*	−0.057	−0.056	−0.209	−0.264*
I59	−0.205	−0.174	−3.301	−2.859	−0.040	−0.072	−0.241	−0.213
L60	−0.216	−0.304*	−2.226	−2.466	−0.022	−0.021	−0.290	−0.259
N61	−0.118	−0.130	.	.	.	.	−0.239	−0.236
N62	−0.112	−0.120	−1.424	−1.325	0.003*	0.019*	−0.267	−0.230
G63	−0.082	0.012	−1.353	−1.248	0.026	−0.010	−0.247	−0.270
H64	.	.	.	.	.	.	.	.
A65	.	.	−1.065	−0.904	.	.	.	.

F66	−0.149	−0.079	−1.196	−1.299	0.011	−0.019	−0.447	−0.395
N67	−0.207	−0.200	−1.580	−1.409	−0.016	−0.026	−0.315	−0.297
V68	−0.346	−0.338	−1.049	−1.224	.	.	−0.412	−0.362
E69	−0.338	−0.482	−1.378	−1.217	−0.056	−0.072	−0.238	−0.217
F70	−0.488	−0.513	−0.708	−0.869	−0.058	−0.075	−0.182	−0.196
D71	−0.433*	−0.157*	−0.648	−0.601	−0.032	−0.086	−0.148	−0.148
D72	−0.343*	−0.426*	−0.425	−0.372	−0.022	−0.011	−0.119	−0.082
S73	0.132	0.068	−0.351	−0.381	−0.010	−0.063	−0.105	−0.141
Q74	0.677*	0.772*	−0.286	−0.331	0.000	0.025	−0.094	−0.109
D75	.	.	−0.176	−0.066*	−0.018	0.010	−0.163	−0.116
K76	.	.	−0.250	−0.229	0.021	0.029	−0.100	−0.105
A77	.	.	−0.259	−0.129	0.018	0.059	−0.115	−0.117
V78	.	.	−0.167	−0.255	0.060	0.004	−0.113	−0.130
L79	.	.	−0.152	−0.144*	−0.022	−0.073	−0.109	−0.101
K80	.	.	−0.074	−0.105	0.012	−0.034	−0.079	−0.112
G81	.	.	−0.066	0.001	−0.010	−0.014	−0.053	−0.076
G82	4.147*	3.804*	−0.027	−0.090	−0.040	−0.037	−0.033	−0.035
L84	2.332*	1.941*	−0.063	−0.037	−0.167	−0.215	−0.050	−0.029
D85	0.929	1.117	−0.069	−0.077	−0.125	−0.042	−0.045	−0.042
G86	1.082	0.737	−0.078	−0.043	−0.050	−0.044	−0.052	−0.036
T87	−1.097*	−0.668*	−0.110	−0.103	−0.024	−0.048	−0.062	−0.097
Y88	0.837*	0.016*	−0.136	−0.101	−0.042	−0.046	−0.083	−0.072
R89	−1.027*	−1.328*	−0.203	−0.246	−0.062	−0.072	−0.098	−0.126
L90	−1.632	−1.318	−0.253	−0.339	−0.049	0.023	−0.134	−0.145
I91	.	.	.	.	.	.	.	.
Q92	−0.415	−0.419	−0.462	−0.524	−0.116	−0.198	−0.199	−0.179
F93	−0.415	−0.433	−0.708	−0.556	−0.099*	−0.161*	−0.267	−0.215
H94	−0.233	−0.182	−0.484	−0.572	−0.173	−0.149	−0.324	−0.306
F95	−0.192*	−0.234*	−0.651	−0.533	−0.030	−0.066	−0.470	−0.451
H96	−0.062	0.024*	−0.310	−0.412	−0.028	0.021	−0.501*	−0.515*
W97	−0.042	−0.055	−0.415	−0.290	0.201	0.214	−0.711	−0.726
G98	−0.061	−0.113	−0.172	−0.265	0.629	0.575	−1.252*	−1.188*
S99	−0.024	−0.041	−0.235	−0.290	0.589	0.495	−0.901*	−0.815*
L100	−0.023	−0.078	−0.133	−0.162	0.781	0.770	−0.786	−0.741
D101	−0.030	−0.057	0.074	−0.006	1.413*	1.407*	.	.
G102	−0.014	−0.032	0.047	0.004	1.506	1.557	−1.441*	−1.016*
Q103	−0.016	0.000	−0.018	−0.084	1.107	0.951	.	.
G104	−0.001	0.050	−0.100	−0.145	0.450	0.500	−0.558*	−0.649*
S105	−0.003	−0.029	−0.076	−0.078	0.361	0.244	−0.356	−0.419
E106	0.005	0.159*	−0.130	−0.148	−0.314	−0.208	−0.238	−0.305
H107	0.092	0.161	−0.101	−0.128	−0.574	−0.601	−0.156	−0.128
T108	0.115	−0.005	−0.054	−0.091	−0.864	−0.890	−0.031	−0.060
V109	0.106	0.072	0.004	−0.047	−1.409*	−1.520*	0.138	0.119
D110	0.155	0.111	0.004	0.003	−1.642*	−1.682*	0.130	0.133
K111	0.108	0.056	−0.008	−0.029	−1.039*	−0.964*	0.102	0.097
K112	0.091	0.053	0.018	−0.019	−1.216*	−0.902*	0.195	0.199
K113	0.046	0.049	0.010	0.006	0.157*	−0.005*	0.155	0.107
Y114	0.026	−0.037	0.000	0.024	.	.	-0.006*	0.082*
A115	−0.014	−0.002	0.109	0.096	.	.	.	.
A116	−0.032	0.012	0.042	0.080	0.856*	0.776*	-0.237*	-0.379*

E117	−0.057	0.011	−0.148	−0.052	0.019	−0.032	−0.469	−0.334
L118	−0.065	−0.056	−0.068	−0.030	−0.585	−0.437	−0.288	−0.303
H119	−0.148	0.039*	−0.299	−0.291	−0.278	−0.396	−0.326	−0.247
L120	−0.065	−0.170	−0.184	−0.148	−0.390	−0.285	−0.194	−0.170
V121	−0.292	−0.275	−0.358	−0.386	−0.183	−0.302	−0.204	−0.177
H122	−0.050	−0.065	−0.244	−0.280	−0.187	−0.221	−0.138	−0.128
W123	−0.528	−0.415	−0.270	−0.232	−0.093	−0.172	−0.113	−0.106
N124	−0.320	−0.516	−0.175	−0.294	−0.107	−0.027	−0.086	−0.091
T125	−1.262	−1.159	−0.151	−0.067	−0.052	−0.062	−0.074	−0.074
K127	−1.193	−1.268	−0.129	−0.072	−0.048	−0.037	−0.063	−0.092
Y128	−0.850	−0.917*	−0.141	−0.074	−0.057	−0.077	−0.062	−0.075
G129	−0.756	−0.625	−0.143	−0.096	−0.039	−0.053	−0.067	−0.042
D130	−0.445	−0.490	−0.163	−0.244	−0.047	−0.058	−0.074	−0.067
F131	−0.359	−0.282	−0.252	−0.231	−0.052	−0.089	−0.085	−0.091
G132	−0.243	−0.236	−0.228	−0.297	−0.055	−0.077	−0.092	−0.094
K133	−0.260	−0.255	−0.174	−0.249	−0.058	−0.122	−0.076	−0.090
A134	−0.311	−0.256	−0.181	−0.178	−0.068	−0.123	−0.081	−0.055
V135	−0.211	−0.230*	−0.199	−0.140	−0.091	−0.111	−0.092	−0.084
Q136	−0.162	−0.089	−0.167	−0.230	−0.091	−0.111	−0.078	−0.092
Q137	−0.140	−0.161	−0.148	−0.195	−0.108	−0.182	−0.076	−0.059
D139	−0.062	−0.046	−0.111	−0.073	−0.140	−0.175	−0.059	−0.084
G140	−0.043	−0.036	−0.134	−0.211	−0.147	−0.157	−0.074	−0.073
L141	0.036	−0.010	−0.159	−0.220	−0.173	−0.234	−0.083	−0.081
A142	0.135	0.295	−0.191	−0.140	−0.191	−0.237	−0.106	−0.089
V143	0.392	0.409	−0.150	−0.214	−0.353	−0.398	−0.109	−0.094
L144	0.175	0.240	−0.172	−0.078	−0.380	−0.464	−0.152	−0.124
G145	0.304	0.127	−0.076	−0.114	−0.831	−0.788	−0.113	−0.110
I146	0.037	0.091	−0.065	0.025	−0.895	−1.081	−0.192	−0.140
F147	0.058	−0.026	0.070	0.036	−2.143*	−1.916*	−0.039	−0.052
L148	−0.024	−0.020	0.106	0.090	1.956*	2.110*	−0.104*	−0.073*
K149	−0.067	−0.021	0.316	0.204	.	.	.	.
V150	−0.051	−0.034	0.321	0.298	.	.	0.655*	0.846*
G151	−0.107	−0.064	0.627	0.669	.	.	.	.
S152	−0.122	−0.099	0.686	0.741	.	.	.	.
A153	−0.178	−0.258	1.219	1.136	.	.	−0.956*	−0.842*
K154	−0.237	−0.256	1.351	1.281	.	.	.	.
G156	.	.	.	.	.	.	.	.
L157	−0.433	−0.458	1.475	1.494	.	.	−0.916*	−0.841*
Q158	−0.403	−0.342	2.423	2.608	−0.360	−0.302	−0.974	−0.971
K159	−0.431	−0.371	3.115	3.731	−0.276	−0.248	−0.687	−0.657
V160	−0.384	−0.366	1.663*	1.813*	.	.	−0.731	−0.665
V161	−0.291	−0.368	.	.	−0.066	−0.036	−0.988*	−0.916*
D162	−0.264	−0.248	.	.	−0.132	−0.084	−0.855	−0.818
V163	−0.236	−0.247	.	.	−0.079	−0.112	−0.608	−0.490
L164	−0.194	−0.182	.	.	−0.012	−0.005	−0.644	−0.557
D165	−0.138	−0.144	.	.	−0.010	0.016	−0.477	−0.417
S166	−0.139	−0.126	.	.	−0.024	−0.030	−0.315	−0.236
I167	−0.136	−0.098	−11.203*	−10.446*	0.039	−0.028	−0.298	−0.274
K168	−0.098	−0.089	−7.403*	−6.021*	0.016	−0.013	−0.204	−0.204
T169	−0.082	−0.077	−3.833	−3.513	0.029	0.020	−0.161	−0.173

K170	−0.063	−0.041	−1.722	−1.836	0.035	0.091	−0.148	−0.180
G171	−0.103	−0.005	−1.681*	−1.393	0.008	−0.012	−0.178	−0.093
K172	−0.080	−0.054	−1.996*	−1.842*	−0.010	−0.030	−0.158	−0.187
S173	−0.089	−0.103	.	.	−0.010	−0.023	−0.152	−0.133
A174	−0.143	−0.075	.	.	−0.025	−0.102	−0.210	−0.129
D175	−0.120	−0.155	.	.	−0.045	−0.088	−0.165	−0.125
F176	−0.224	−0.314	.	.	−0.052	−0.081	−0.195	−0.248
T177	−0.283	−0.349	.	.	−0.073	−0.081	−0.205	−0.210
N178	−0.481	−0.557	−1.283	−1.278	−0.051	−0.085	−0.184	−0.225
F179	−0.703	−0.747	−0.885	−0.618	−0.069	−0.104	−0.238	−0.264
D180	−1.028	−1.191	0.139	−0.073	−0.106	−0.114	−0.305	−0.305
R182	−1.995	−1.801	0.085	0.138	0.024	0.009	−0.248	−0.209
G183	−1.296	−1.218	0.307	0.410	−0.079	−0.068	−0.319	−0.334
L184	−0.901	−0.769	0.281	0.349	−0.163	−0.179	−0.344	−0.401
L185	−0.757	−0.580	0.281	0.195	−0.167	−0.333	−0.290	−0.273
E187	−0.057	−0.028	0.260	0.196	.	.	−0.007	−0.001
S188	0.126	0.130	0.188	0.144	.	.	−0.018	−0.025
L189	0.471*	0.425*	0.099	0.108	0.927	0.614	−0.025	−0.012
D190	0.558*	0.611*	0.005	0.059	−0.297*	−0.527*	0.014	−0.014
Y191	0.831	0.749	0.019	0.061	−1.046*	−1.063*	.	.
W192	0.567	0.608	0.008	−0.074	−1.559	−1.547	0.002	0.033
T193	0.591	0.646	−0.030	0.025	−0.865	−0.864	−0.006	0.015
Y194	0.502	0.551	−0.070	−0.117	−0.596	−0.678	−0.046	−0.019
G196	0.225	0.220	−0.116	−0.186	−0.335	−0.291	−0.072	−0.088
S197	0.123	0.278*	−0.150	0.049*	−0.306	−0.240	−0.085	0.028*
L198	−0.011*	−0.044*	−0.194	−0.245	−0.188	−0.259	−0.116	−0.154
T199	.	.	.	.	.	.	.	.
T200	.	.	−0.305	−0.339	−0.142	−0.028	−0.182	−0.177
L203	0.002	0.136*	−0.257	−0.291	−0.111	−0.212	−0.135	−0.118
L204	−0.028	−0.078	−0.217	−0.216	−0.130	−0.176	−0.113	−0.071
E205	−0.004	−0.035	−0.151	−0.119	−0.145	−0.167	−0.083	−0.095
C206	0.031	0.032	−0.163*	−0.333*	−0.191	−0.150	−0.092	−0.097
V207	0.108	0.155	−0.151	−0.232	−0.232	−0.209	−0.085	−0.085
T208	0.307	0.385	−0.137	−0.110	−0.275	−0.329	−0.085	−0.063
W209	0.418	0.469	−0.101	−0.186	−0.456	−0.513	−0.067	−0.061
I210	0.532	0.659	−0.101	−0.045	−0.595	−0.632	−0.091	−0.042
V211	0.540	0.459	−0.038	−0.074	−1.062	−1.108	−0.040	−0.032
L212	0.328	0.495	−0.003	0.071	−1.656	−1.761	−0.056	0.002
K213	0.416	0.326	0.061	0.118	.	.	0.042	0.112
E214	0.260	0.210	0.115	0.142	.	.	0.071	0.073
I216	−0.061	−0.102	0.248	0.234	.	.	−0.022	−0.018
S217	−0.152	−0.155	0.514	0.429	.	.	.	.
V218	−0.131	−0.103	0.585	0.658	.	.	.	.
S219	−0.184	−0.159	1.143	1.136	.	.	.	.
S220	−0.108	−0.112	0.850	0.897	1.370*	1.267*	.	.
E221	−0.121	−0.103	1.191	1.196	0.539*	0.595*	.	.
Q222	−0.150	−0.122	1.345	1.563	0.504	0.395	.	.
V223	−0.145	−0.160	0.760	0.718	0.818*	0.603*	.	.
L224	−0.114	−0.148	0.570	0.422	0.652	0.620	.	.
K225	−0.131	−0.121	0.523	0.347	0.346	0.338	.	.

F226	−0.130	−0.130	−0.144	−0.368	0.349	0.269	.	.
R227	−0.103	−0.160	−0.466	−0.663	0.372	0.315	.	.
K228	−0.098	−0.129	−1.091	−1.159	0.243	0.322	.	.
L229	−0.095	−0.037	−1.652	−1.877	0.183	0.138	−0.632*	−0.467*
N230	−0.080	−0.064	−2.396	−2.143	0.093	0.151	−0.282	−0.309
F231	−0.060	−0.052	−1.234	−1.323	0.108	0.129	−0.231	−0.311
N232	−0.046	−0.031	−1.250	−1.151	0.079	0.035	−0.143	−0.169
G233	−0.047	−0.072	−0.987	−1.209	0.039	0.039	−0.074	−0.081
E234	−0.046	−0.057	−1.285	−1.276	0.020	0.097	−0.051	−0.054
G235	−0.033	−0.028	−1.229	−1.090*	0.034	0.014	0.031	0.023
E236	−0.037	−0.069	−0.983	−0.932	0.044	0.099	0.011	0.019
E238	−0.027	−0.055	−0.932	−1.002	0.093	0.110	0.118	0.109
E239	−0.049	−0.027	−1.108	−1.012	0.105	0.129	−0.053	−0.047
L240	−0.043	−0.075	−0.858	−1.013	0.172	0.236	−0.154	−0.224
M241	−0.066	−0.006	−1.149	−1.162	0.169	0.075	−0.561	−0.437
V242	−0.053	−0.068	−0.708	−0.686	0.215	0.253	−0.610	−0.706
D243	−0.055	−0.072	−0.412	−0.484	0.349	0.341	−0.871	−0.837
N244	−0.040	−0.042	−0.450	−0.485	0.193	0.111	−0.663	−0.620
W245	−0.027	−0.135	−0.344	−0.395	0.107	0.136	−0.487	−0.474
R246	−0.022	−0.052	−0.264	−0.326	−0.089*	−0.042*	−0.303	−0.332
A248	0.051	0.023	−0.121	−0.187	−0.205	−0.213	−0.163	−0.193
Q249	0.105	0.071	−0.096	−0.171	−0.280	−0.268	−0.100	−0.094
L251	0.112	0.121	−0.068	−0.082	−0.385	−0.363	−0.041	−0.084
K252	0.098	0.071	−0.058	−0.084	−0.321	−0.292	−0.029	−0.049
N253	0.118	0.114	−0.060	−0.014	−0.304	−0.353	−0.028	−0.016
R254	0.155	0.157	−0.052	−0.089	−0.362	−0.276	−0.023	−0.022
Q255	0.248	0.232	−0.048	0.012	−0.431	−0.477	−0.012	−0.024
I256	0.287	0.347	−0.028	−0.041	−0.666	−0.692	0.007	0.011
K257	0.477	0.450	−0.034	0.065	−0.729	−0.779	−0.002	0.037
A258	0.539	0.559	−0.006	−0.043	−0.830	−0.959	0.027	0.012
S259	0.759	0.727	0.011	−0.008	−0.973	−0.809	0.021	0.032
F260	0.655	0.673	0.023	0.004	−0.985	−0.786	0.047	0.051
K261	0.542	0.524	0.047	−0.034	−0.633	−0.785	0.066	0.066

PCS values marked with an asterisk (*) originate from HN or N nuclei that are closer than 15 Å to the Tm metal and were excluded in the Numbat fit.
